# Metagenomic Analysis Reveals Microbial Interactions at the Biocathode of a Bioelectrochemical System Capable of Simultaneous Trichloroethylene and Cr(VI) Reduction

**DOI:** 10.3389/fmicb.2021.747670

**Published:** 2021-09-30

**Authors:** Bruna Matturro, Marco Zeppilli, Agnese Lai, Mauro Majone, Simona Rossetti

**Affiliations:** ^1^Water Research Institute, IRSA-CNR, Rome, Italy; ^2^Department of Chemistry, Sapienza University of Rome, Rome, Italy

**Keywords:** reductive dechlorination, Cr(VI) reduction, bioelectrochemical remediation, *Dehalococcoides mccartyi*, *Methanobacterium formicicum*, *Methanobrevibacter arboriphilus*

## Abstract

Bioelectrochemical systems (BES) are attractive and versatile options for the bioremediation of organic or inorganic pollutants, including trichloroethylene (TCE) and Cr(VI), often found as co-contaminants in the environment. The elucidation of the microbial players’ role in the bioelectroremediation processes for treating multicontaminated groundwater is still a research need that attracts scientific interest. In this study, 16S rRNA gene amplicon sequencing and whole shotgun metagenomics revealed the leading microbial players and the primary metabolic interactions occurring in the biofilm growing at the biocathode where TCE reductive dechlorination (RD), hydrogenotrophic methanogenesis, and Cr(VI) reduction occurred. The presence of Cr(VI) did not negatively affect the TCE degradation, as evidenced by the RD rates estimated during the reactor operation with TCE (111±2 μeq/Ld) and TCE/Cr(VI) (146±2 μeq/Ld). Accordingly, *Dehalococcoides mccartyi*, the primary biomarker of the RD process, was found on the biocathode treating both TCE (7.82E+04±2.9E+04 16S rRNA gene copies g^−1^ graphite) and TCE/Cr(VI) (3.2E+07±2.37E+0716S rRNA gene copies g^−1^ graphite) contamination. The metagenomic analysis revealed a selected microbial consortium on the TCE/Cr(VI) biocathode. *D. mccartyi* was the sole dechlorinating microbe with H_2_ uptake as the only electron supply mechanism, suggesting that electroactivity is not a property of this microorganism. *Methanobrevibacter arboriphilus* and *Methanobacterium formicicum* also colonized the biocathode as H_2_ consumers for the CH_4_ production and cofactor suppliers for *D. mccartyi* cobalamin biosynthesis. Interestingly, *M. formicicum* also harbors gene complexes involved in the Cr(VI) reduction through extracellular and intracellular mechanisms.

## Introduction

Trichloroethylene (TCE) is a toxic and persistent anthropogenic pollutant commonly found in groundwater and often detected in the aquifer with other co-contaminants, including heavy metals such as the carcinogenic Cr(VI) (Watts et al., 2015). Despite the persistence into the environment, TCE is a biodegradable compound, while Cr(VI) can be transformed into less toxic forms (i.e., Cr(III)).

The TCE transformation to cis-1,2-dichloroethene (cis-DCE) and vinyl chloride (VC) up to the harmless ethene occurs *via* the anaerobic reductive dechlorination (RD) in the presence of an electron donor and through the biological activity of specialized organohalide-respiring bacteria (OHRB; Strycharz et al., 2008; Löffler et al., 2013). Among them, *Dehalococcoides mccartyi* is the sole microorganism capable of performing RD to ethene. Thus, it is considered a robust biomarker for the RD monitoring and evaluation at laboratory and field scale (Taş et al., 2010; Saiyari et al., 2018). *Dehalococcoides mccartyi* strains are characterized by electron transport chain’s essential membrane-associated enzymes, known as reductive dehalogenases (RDases; Ahsanul Islam et al., 2010; Pérez-de-Mora et al., 2018; Hermon et al., 2019). Some RDases have been functionally characterized (i.e., *TceA*, *VcrA*, and *BvcA*), but most of them remain uncharacterized (RdhAses) among dechlorinating isolates or mixed cultures (Hug et al., 2013; Molenda et al., 2020). *Dehalococcoides mccartyi* is strictly anaerobic and sensitive to oxygen exposure, incapable of substrate fermentation as a source of electrons, and unable to synthesize corrinoids (i.e., cobalamin), fundamental cofactors for the functionality of RDases (Yan et al., 2016; Yang et al., 2017). These metabolic peculiarities make *D. mccartyi* growth and RD activity favored within mixed anaerobic consortia where non-dechlorinating microorganisms can supply exogenous cofactors.

In respect of Cr(VI) remediation, anaerobic microorganisms capable of Cr(VI) to Cr(III) reduction (i.e., *Pseudomonas dechloromaticans*, *Enterobacter cloacae*, *Shewanella oneidensis*, and *Clostridium chromiireducens*) and with Cr(VI) tolerance (i.e., *Bacillus* sp., *Leucobacter* sp., *Exiguobacterium* sp., *Microcococcus* sp., *Rhodococcus* sp., *Arthrobacter* sp., *Achromobacter* sp., and *Ochrobactrum* sp.) have been reported (Horitsu et al., 1987; Turick and Apel, 1997; Desai et al., 2008; Sarangi and Krishnan, 2008; Elangovan et al., 2010; Beretta et al., 2018; Kabir et al., 2018). Several studies also demonstrated that indirect Cr(VI) bioreduction is achievable through injections of biodegradable organic substrates that prompt anaerobic conditions and reductant production such as iron and sulfur species capable of mediating Cr(VI) reduction to Cr(III) (Kim et al., 2001; Somenahally et al., 2013; Beretta et al., 2018, 2019). Further, under anaerobic conditions, direct Cr(VI) reduction can be mediated by membrane-bound reductases encoded by *mtrA*, *mtrB*, *mtrC* genes, and soluble enzymes (e.g., soluble cytochrome c3; Belchik et al., 2011; Thatoi et al., 2014). Some studies also reported that Cr(VI) reduction occurs with H_2_ as electron donor and CO_2_ as a carbon source in anaerobic mixed cultures (Marsh and McInerney, 2001).

The simultaneous biodegradation of TCE and biotransformation of Cr(VI) to the less toxic form Cr(III) is a challenging remediation goal when co-contamination occurs. However, the available bioremediation technologies commonly treat TCE or Cr(VI) separately (Malaviya and Singh, 2011; Shukla et al., 2014; Jin et al., 2015; Sophia and Saikant, 2016; Wang et al., 2020). Among these, bioelectrochemical technologies emerged in the last years as the most attractive and versatile options being advantageous in terms of cost-effectiveness, side reactions control, and sustainability for *in situ* remediation of contaminated matrices (Fernando et al., 2019; Cecconet et al., 2020; Wang et al., 2020). Recently, there has been an ever-increasing interest in biocatalyzed reduction at the cathode (i.e., microbial electrolysis cells, MECs) of many pollutants, including TCE or Cr(VI) (Palma et al., 2018). These systems require an electrode acting as an electron donor (cathode), an electron sink (anode), and microorganisms driving reactions to exploit energy from toxic contaminants (Modin and Aulenta, 2017; Ivase et al., 2020). Several studies have demonstrated that MECs, under controlled potentials, may rapidly enhance RD by establishing dechlorinating consortia growing on the biocathode (Aulenta et al., 2007, 2011; Verdini et al., 2015; Zeppilli et al., 2019). Nevertheless, despite various aspects of MECs for TCE dechlorination have been investigated (i.e., the effect of cathode potential on TCE dechlorination rate, selectivity, electron transfer mechanisms; Aulenta et al., 2007, 2008, 2010, 2011; Chen et al., 2018; Wang et al., 2020), only a few reports documented the microbial composition of the TCE-reducing biocathode without the addition of external carbon substrates (Aulenta et al., 2010; Di Battista et al., 2012; Wang et al., 2015) and even less in the case of TCE/Cr(VI) co-contamination. Similarly, very little is known about the microbial players of the bioelectrochemical systems (BES) treating Cr(VI) contamination. A recent study conducted at the Cr(VI)-reducing biocathode revealed γ-*Proteobacteria* as the most abundant electrotrophic component and, among them, also the presence of known chromium reducing/resistant bacteria including *Pseudomonas* sp. and *Ochrobactrum* sp. (Romo et al., 2019). Pieces of evidence reported that the production of H_2_ at the cathode of a bioelectrochemical system could favor the autotrophic reduction of Cr(VI) by hydrogenotrophic bacteria.

Therefore, despite that the BES treating TCE or Cr(VI) have been previously described along with some biological data reporting the microbial players involved in the reductive processes, the feasibility of a bioelectrochemical system treating TCE in the presence of Cr(VI) as co-contaminants has been only recently investigated (Lai et al., 2021). Nonetheless, the microbial composition and interactions occurring at the biocathode treating TCE/Cr(VI) co-contamination still need to be analyzed. To this aim, metagenomics approaches provide reliable information about the key enzymes/genes involved in the degradation and detoxification of environmental pollutants to understand better the microbial community structure, functions, and interactions in contaminated matrices or engineered systems for the biological removal of pollutants, with implications for process optimization and bioremediation application at field scale (Bharagava et al., 2019; Lapidus and Korobeynikov, 2021). Metagenomic studies have been previously conducted on stable dechlorinating consortia to shed light on the metabolic features of the microbial components and their role in the RD processes under different conditions (Brisson et al., 2012; Maphosa et al., 2012; Dam et al., 2017; Wang et al., 2019; Kucharzyk et al., 2020). Similarly, some metagenomic studies have also been conducted on biological systems for Cr(VI) removal to explore the metabolic potential, including Cr(VI) remediation genes, primarily in wastewater treatment systems or microbial consortia (Miao et al., 2015; Sun et al., 2019; Pei et al., 2020; Rahman and Thomas, 2021). To the best of our knowledge, no metagenomic studies have been conducted on a bioelectrochemical system for simultaneous TCE/Cr(VI) reduction.

In this study, the biofilm growing on the biocathode of a bioelectrochemical system capable of complete TCE-to-VC/ethene reduction in the presence of Cr(VI) as a co-contaminant has been characterized for the first time. The RD process biomarkers, including *D. mccartyi* 16S rRNA and RDase genes, have been monitored. The metagenomic analysis has been performed to define the microbiome composition and metabolic features of the critical biological players of the processes occurring in the bioelectrochemical system.

## Materials and Methods

### Continuous Flow Reactor: Set Up and Operating Conditions

**Figure 1:**
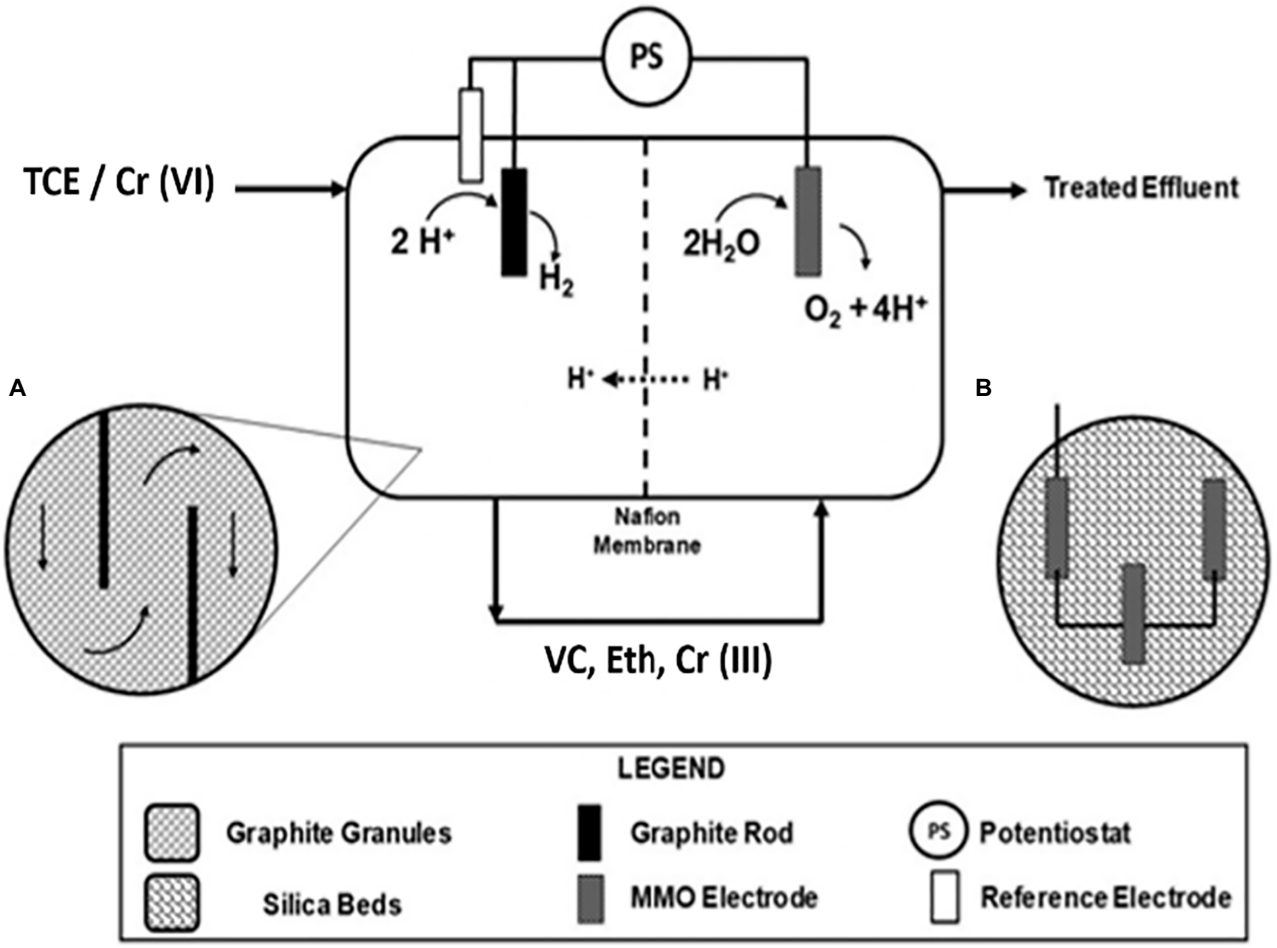
Scheme of the continuous flow bioelectrochemical reactor for the trichloroethylene (TCE) and Cr(VI) removal. Horizontal cross section detail of the cathodic chamber **(A)** and schematic representation of the MMO (Magneto special Anodes, Netherland) electrode inserted in the silica bed **(B)**.

The continuous flow bioelectrochemical reactor scheme for the TCE and Cr(VI) removal used in this study is reported in Figure 1. The continuous flow bioelectrochemical reactor was composed of a 0.821L cathodic chamber (Figure 1A) filled with graphite granules (2 and 6mm; El Carb 100, Graphite sales, Inc., United States). The cathodic chamber adopted a graphite rod as a current collector and a 0.90L anodic chamber composed of an MMO Electrode (Magneto special Anodes, Netherland) inserted in a silica bed (Figure 1B).

An anaerobic mineral medium (NH_4_Cl, 0.5; MgCl_2_x6H_2_O, 0.1; CaCl_2_x2H_2_O, 0.05; K_2_HPO_4_, 0.4gL^−1^) supplemented with a metal and vitamin solution was used as previously described (Lai et al., 2017). The cathode and anode chambers were physically separated by the Nafion® 117 proton exchange membrane, which allowed the proton migration to maintain electroneutrality. An Ag/AgCl reference electrode (+0.199 vs. standard hydrogen electrode, SHE; Amel, Milan, Italy) placed in the cathode chamber permitted the polarization of the reactor by a potentiostat (Amel Model 549, Milan) adopting a three-electrode configuration. Two Teflon® septa were inserted within the graphite granules in the cathodic chamber to obligate the flow direction along the total cathodic volume (Figure 1). The cathodic chamber was inoculated with a hydrogenophilic dechlorinating, while the anodic chamber was inoculated with an anaerobic dechlorinating mixed culture (Lai et al., 2021). The continuous flow reactor was operated with the TCE-contaminated mineral medium (TCE 50μM) and the TCE/Cr(VI)-contaminated mineral medium (Lai et al., 2021). The flow rate was 0.58l/d, corresponding to a cathodic hydraulic retention time (HRT) of 1.4day. The cathodic chamber of the bioelectrochemical reactor was polarized at −650mV vs. SHE. The average rates of RD (1) and Cr(VI) reduction (2) were calculated as follows:


$$r_{RD}\;\left[\frac{\mu eq}{Ld}\right]=\frac{\left(2x\left[cisDCE\right]+4x\left[VC\right]+6x\left[ETH\right]+8x\left[ETA\right]\right)\;x\;Q}{V_{c}}$$ (1)

$$r_{Cr\left(VI\right)}\;\left[\frac{\mu eq}{Ld}\right]=\frac{\left(3x\left[Cr\left(VI\right)\right]\right)\;x\;Q}{V_{c}}$$ (2)

where [cisDCE], [VC], [ETH], and [ETA] are the average liquid-phase compound concentration (μM); 2, 4, 6, and 8 are the number of moles of electrons required for the formation of RD intermediates from 1mol of TCE, while 3 is the equivalent moles required for the reduction of Cr(VI) to Cr(III); Q is the flow rate (L d^−1^); VC is the empty volume of the cathode chamber (L) (i.e., the volume without the electrode).

The Coulombic efficiency (CE_RD_) has been calculated as follows (3):


$$CE_{RD}\left(\%\right)=\frac{\frac{r_{i}x\;V_{c}\;}{24x3600}\mathrm{xF}}{I}\;x\;100$$ (3)

where r_i_ is the average rate of the formation of RD compounds or reduction of Cr(VI) (μM d^−1^), F is the Faraday’s constant (96,485 C mol eq^−1^), and I is the electric current (μA; Lai et al., 2021). Methane production rate (4) and its relative contribution to the current consumption (i.e., Coulombic efficiency for methane generation) (4) were assessed as follows (Zeppilli et al., 2019, 2020):


$$CH_{4}\;\left(mA\right)=Q\left(\;\frac{L}{d}\right)*8\left[CH_{4}\right]*\frac{F}{\frac{s}{d}}$$ (4)

$$CE_{CH4}\;\left(\%\right)=\frac{CH_{4\left(mA\right)}}{Current\;\left(mA\right)}*100$$ (5)

where Q is the liquid flow rate, [CH_4_] represents the liquid methane concentration calculated according to the Henry’s law constant at room temperature, F is the Faraday constant (i.e., 96,485 C/mol e^−^), and s/d represents the seconds in a day (86,400s/day).

### Sampling for Biomolecular Analysis and DNA Extraction

Samples for biomolecular analysis were collected in triplicate from the biocathode treating TCE only (sample hereafter cited as TCE) and TCE/Cr(VI) as co-contaminants (sample hereafter cited as TCE/Cr(VI)). One gram of biocathode (i.e., graphite granules) was collected from TCE and TCE/Cr(VI) samples. DNA extraction was performed with DNeasy PowerSoil Kit (Qiagen, Italy), following the manufacturer’s instructions. Purified DNA from each sample was eluted in 100μl sterile Milli-Q and stored at −20°C until further biomolecular analysis.

### Real-Time PCR

*Dehalococcoides mccartyi* 16S rRNA gene and reductive dehalogenase genes *tceA*, *bvcA*, *vcrA* were quantified *via* absolute quantification qPCR assays. TaqMan® chemistry (6-carboxyfluorescein-FAM at the 5' end as reporter fluorophore; N, N, N, N,-tetramethyl-6-carboxyrhodamine-TAMRA at the 3' end as a quencher) was employed, and reactions were conducted in 20μl total volume including 3μl of template DNA, 300nM of each primer, 300nM TaqMan® probe and SsoAdvancedTM Universal Probes Supermix (Bio-Rad, Italy). Primers and probes used were previously reported (Ritalahti et al., 2006). Standard curves for the absolute quantification were constructed with the long PCR amplicons of the targeted genes (Matturro et al., 2013). Reactions were run in triplicate for each biological sample, and qPCR was performed with the CFX96 TouchTM Real-Time PCR Detection System (Bio-Rad, Italy). Quantitative data are reported as gene copy numbers g^−1^ of graphite granules.

### Sequencing

The whole microbiome composition of the TCE and TCE/Cr(VI) biocathodes was analyzed by 16S rRNA gene amplicon sequencing. The biomolecular analysis was expanded through whole shotgun metagenomic sequencing and a genome-centric analysis. A *de novo* metagenome assembly was performed on quality-filtered reads, and individual genomes (genome bins) were subsequently extracted from the assembled metagenome using a customized bioinformatics approach. The individual bins were subjected to quality assessment, taxonomic classification, and gene annotation. In the following, the detailed methods for 16S rRNA gene and shotgun metagenomic sequencing are reported.

#### 16S rRNA Gene Amplicon Sequencing and Bioinformatics

Four nanograms of DNA extracted from TCE and TCE/Cr(VI) biocathode was used for NGS analysis. DNA extraction 16S rRNA Amplicon Library Preparation targeting the V1–3 variable region was constructed as previously reported (Matturro et al., 2017). PCR reactions were performed in 25μl total volume containing Phusion Master Mix High Fidelity (Thermo Fisher Scientific, United States) and 0.5μM final concentration of the library adaptors with V1–V3 primers (Bacteria, 27F: 5'-AGAGTTTGATCCTGGCTCAG-3'; 534R: 5'-ATTACCGCGGCTGCTGG-3') and V3-V5 primers (Archaea, 340F: 5'-CCCTAHGGGGYGCASCA-3'; 915R: 5'-GWGCYCCCCCGYCAATTC-3'). All PCR reactions were run in duplicate and pooled afterward. Libraries were purified using the Agencourt® AMpure XP-beads protocol (Beckman Coulter, Italy), and the concentration was measured with Qubit 3.0 Fluorometer (Thermo Fisher Scientific, Italy). Purified libraries were pooled in equimolar concentrations and diluted to 4nM. PhiX control (15%) was added at 10% in the pooled libraries to overcome issues often observed in 16S rRNA gene sequencing. Samples were paired-end sequenced (2×301bp) on a MiSeq (Illumina, United States) instrument using a MiSeq Reagent kit v3, 600cycles (Illumina, United States) following the standard guidelines. Raw data were processed and analyzed using QIIME2 software tools 2018.2 release (c). The reads were demultiplexed using demux plugin1, denoised, dereplicated, and chimera-filtered using the DADA2 algorithm (Callahan et al., 2016). The taxonomic analysis was based on a Naïve Bayes classifier trained on 16S rRNA gene sequences clustered at 99% similarities within the Silva 132–99 database (release December 2017),^1^ allowing to construct a data set of amplicon sequence variants (ASVs).

#### Metagenomic Sequencing and Bioinformatics

Sample DNA concentrations from TCE/Cr(VI) contaminated biocathode were measured using the Qubit dsDNA HS kit. The DNA quality and concentrations were evaluated using TapeStation with the Genomic ScreenTape (Agilent Technologies). The final concentration of 1.5ng/μl has been used for the library preparation. Sequencing libraries were prepared using the NEB Next Ultra II DNA library prep kit for Illumina (New England Biolabs, United States) following the manufacturer’s protocol. Library concentrations were measured in triplicate using the Qubit dsDNA HS kit and library size estimated using TapeStation with D1000 HS ScreenTape. The sequencing libraries were pooled in equimolar concentrations and diluted to 4nM. The samples were paired-end sequenced (2x301bp) on a MiSeq (Illumina, United States) using a MiSeq Reagent kit v3, 600cycles (Illumina, United States) following the standard guidelines for preparing and loading samples on the MiSeq. Raw Illumina reads were filtered for PhiX using Usearch11 (Edgar, 2010) subsequently trimmed using Cutadapt v. 2.10 (Martin, 2011). Forward and reverse reads were used to perform *de novo* assembly in megahit v. 1.2.9. The total assembly length of the metagenomes, the length of the longest contig, and the shortest contig length needed to cover 50% of the genome were calculated. Individual genomes (genome bins) were subsequently extracted from each sample metagenome in mmgenome2 v. 2.1.3. Bins were quality assessed with CheckM v. 1.1.3 (Parks et al., 2015). Classification of bacterial bins was conducted with the Genome Taxonomy Database toolkit (GTDB-TK) v. 1.3.0 (Chaumeil et al., 2019). Average nucleotide identities (ANI) were calculated using FastANI v. 1.32 (Goris et al., 2007; Jain et al., 2018). Genome annotations of bacterial and archaeal genomes were firstly conducted with Prokka v. 1.14.6. Further, NCBI Prokaryotic Genome Annotation Pipeline (PGAP) was also performed for the deepest annotation. The Whole Genome Shotgun project was deposited at DDBJ/ENA/GenBank under the multiple accession JADIIK000000000-JADIIN000000000. Raw data are deposited under the SRA accession SRR12879946 within the BioProject PRJNA670625.

## Results

### Performance of the Bioelectrochemical Reactor Operating With TCE and TCE/Cr(VI) Contamination

**Figure 2:**
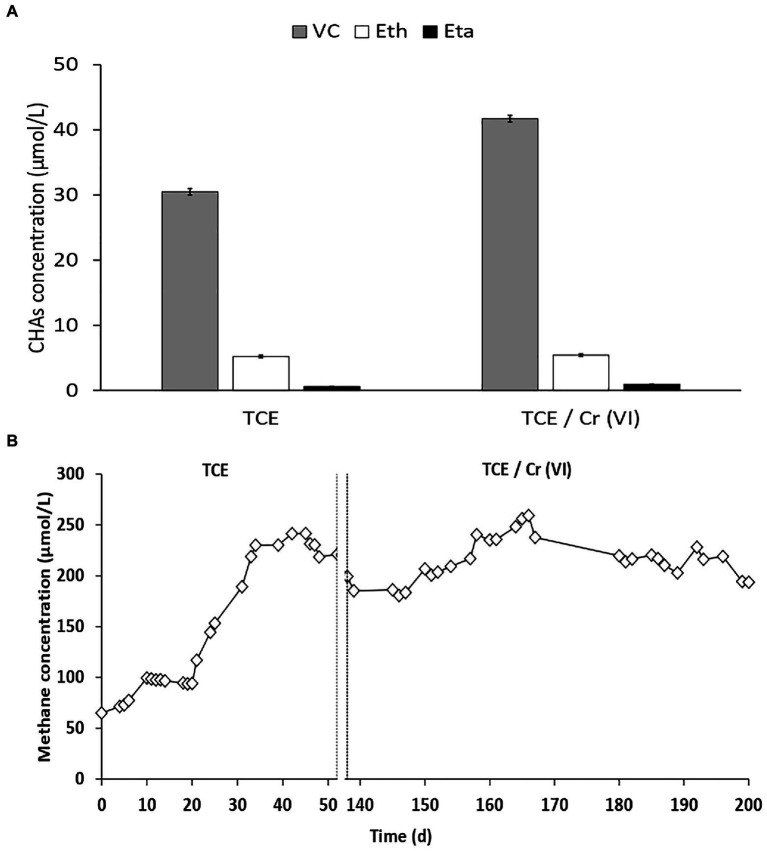
**(A)** Distribution of the reductive dechlorination (RD) products observed in the cathode outlet TCE and TCE/Cr(VI) operating conditions. **(B)** CH_4_ concentrations in the cathodic effluent of the bioelectrochemical reactor during all the system’s operation periods, including the runs with TCE and TCE/Cr(VI).

In the cathodic chamber under continuous flow, the complete TCE removal occurred also in the presence of Cr(VI) as co-contaminant. The dechlorination products observed in the cathodic chamber fed with TCE and TCE/Cr(VI) were VC, ethene, and ethane (Figure 2). RD rates obtained during TCE and TCE/Cr(VI) operating conditions were 111±2 and 146±2 μeq/Ld, respectively (Table 1).

**Table 1:** Reductive dechlorination and methanogenesis performances during TCE and TCE/Cr(VI) operating conditions.

	TCE^*^	TCE/Cr(VI)^*^
TCE load rate (μmol/Ld)	26±5	38±2
TCE removal efficiency	100±1	100±3
RD (μeq/Ld)	111±2	146±2
CE-RD (%)	2.7±0.1	4.4±0.5
CH_4_ production rate (μeq/Ld)	1,301±18	1,224±30
CE-CH_4_ (%)	31±2	39±1
Cr(VI) removal efficiency	0	100
Cr(VI) reduction rate (μeq/Ld)	0	91.1±5.2
CE-Cr(VI) (%)	0	2.72±0.19
CE-excess (%)	66.3	53.88

CE, Coulombic efficiency and RD, reductive dechlorination.

* Data previously reported in Lai et al. (2021).

The Coulombic efficiency (i.e., the electrons utilized by the dehalorespiring bacteria to reduce the chlorinated compounds, CE-RD) allowed for the consumption of 2.7±0.1 and 4.4±0.5% of the electric current. Bioelectrochemical CO_2_ reduction into CH_4_ (i.e., bioelectromethanogenesis) was observed in the cathodic chamber during all the operating periods with a constant concentration of 231±1μmol/L (Figure 2B). In particular, during the run with TCE and with TCE/Cr(VI), similar CH_4_ production rates were observed with average values of 1,301±18 and 1,224±30 μeq/Ld, respectively (Table 1). These CH_4_ production rates corresponded to the consumption of 31 and 39% of the electrical current generated by the bioelectrochemical cell in the TCE and TCE/Cr(VI) runs, respectively. Moreover, Cr(VI) was completely removed with a reduction rate of 91.1±5.2 μeq/Ld and Coulombic efficiencies (CE-Cr(VI)) of 2.72±0.19%. Overall, the CE excess estimated in the TCE-fed and TCE/Cr(VI) fed bioreactor was 66.3 and 53.8%, respectively.

### Microbiome Composition of the TCE and TCE/Cr(VI) Cathodic Biofilms

**Figure 3:**
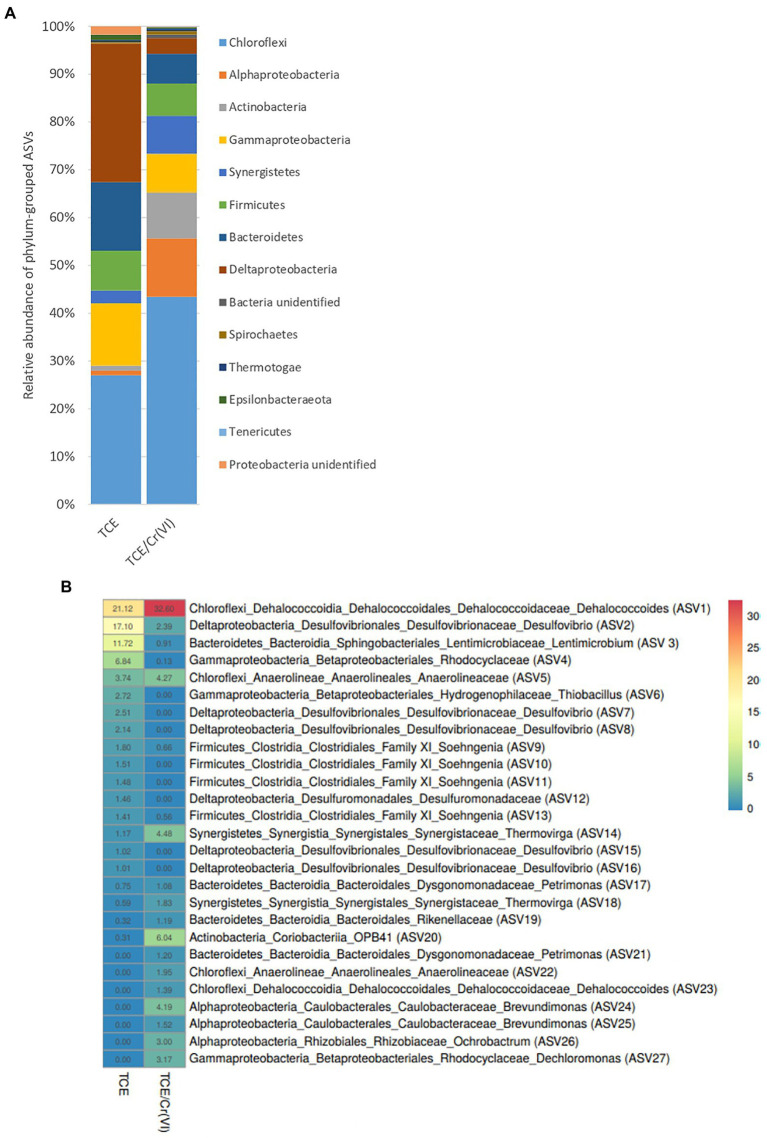
Microbiome composition of the cathodic biofilm at phylum level **(A)** and heatmap of the most abundant ASVs at genus level **(B)** found in the biofilm of the bioelectrochemical reactor treating TCE and TCE/Cr(VI).

**Figure 4:**
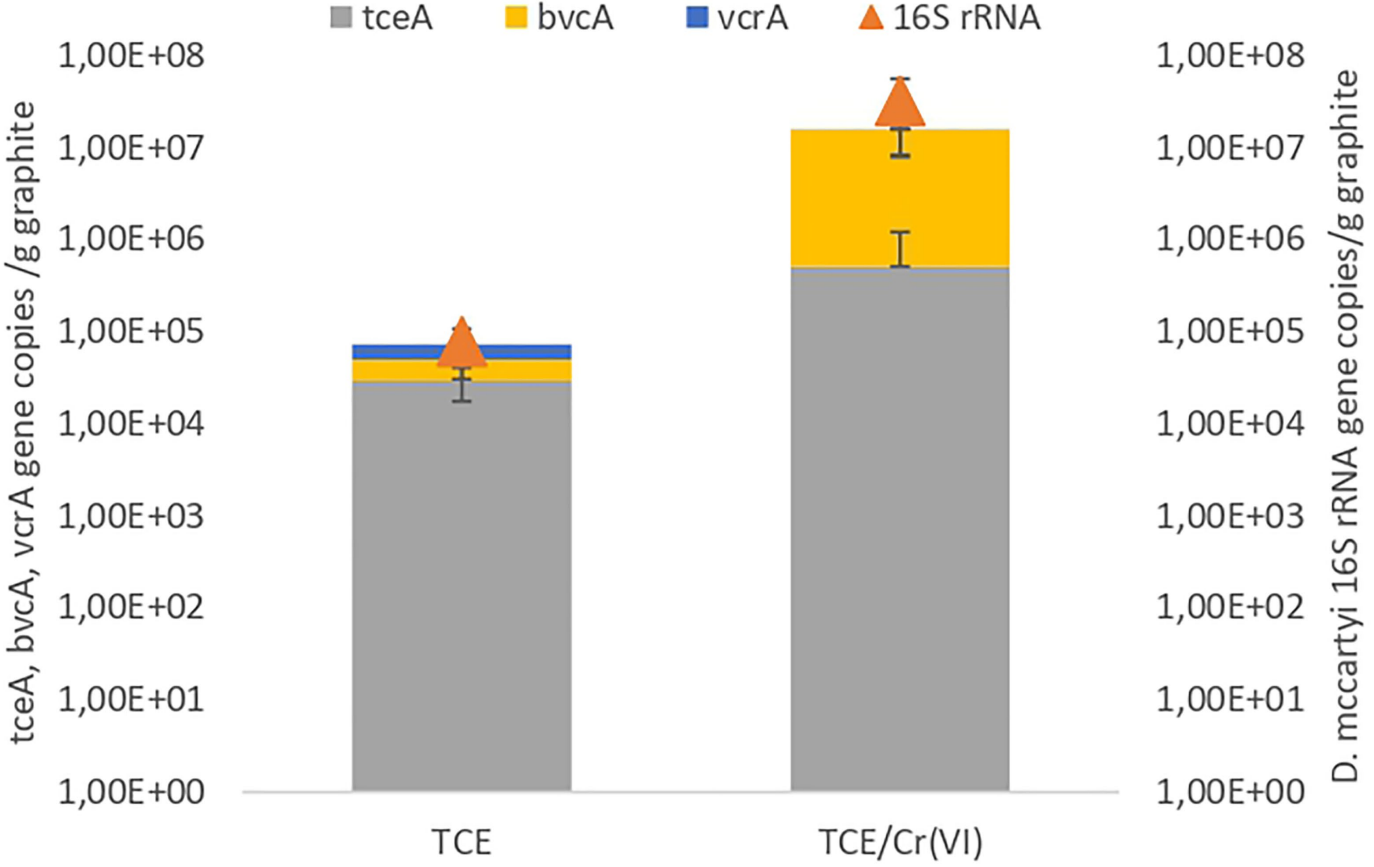
Quantification of 16S rRNA of *Dehalococcoides mccartyi* and strain-specific RDase genes conducted on the samples from the operating conditions with TCE and TCE/Cr(VI). Data are plotted in log scale (base 10).

The microbiome composition of the TCE and TCE/Cr(VI) biocathode, including *Bacteria* and *Archaea*, was firstly characterized through 16S rRNA gene amplicon sequencing (Figure 3A). On the TCE biocathode, members of *Chloroflexi* phylum (27%), including mostly *D. mccartyi* (21.12%), and *Deltaproteobacteria* (28.9%) affiliated to *Desulfovibrio* (27%), were mainly found. In particular, *D. mccartyi* detected on the biocathode of the bioelectrochemical reactor running with TCE accounted for 7.82E+04±2.9E+04 16S rRNA gene copies g^−1^ graphite and were mostly tceA-carrying strains (Figure 4). *Bacteroidetes* phylum represented 14% of total ASVs and included *Lentimicrobium* species (12%). *Gammaproteobacteria* also colonized the biocathode (13%), including 7% of *Rhodocyclaceae* and 3% of *Thiobacillus* (Figure 3B).

Similarly, the TCE/Cr(VI) biofilm was mainly composed of *Chloroflexi* (43%), including 35.18% of *D. mccartyi* (Figure 3A), the latter accounted for 3.2E+07±2.37E+07 16S rRNA gene copies g^−1^ graphite, mostly tceA-carrying strains (Figure 4). Moreover, *Alphaproteobacteria* (12%) were detected and included *Brevundimonas* (6%) and *Ochrobactrum* species (4%), while *Gammaproteobacteria* (8%) included *Thiobacillus* (1%), *Dechlomononas* (3.2%), and unidentified *Rhodocyclaceae* (2%). At a lower extent, *Thermovirga* of the phylum *Synergistetes* (8%), *Soehngenia* (2.4%), and *Acidaminobacter* (1.8%) of the phylum *Firmicutes* (7%) and *Desulfovibrio* (2.6%) within *Deltaproteobacteria* (3%) were found (Figure 3A).

**Figure 5:**
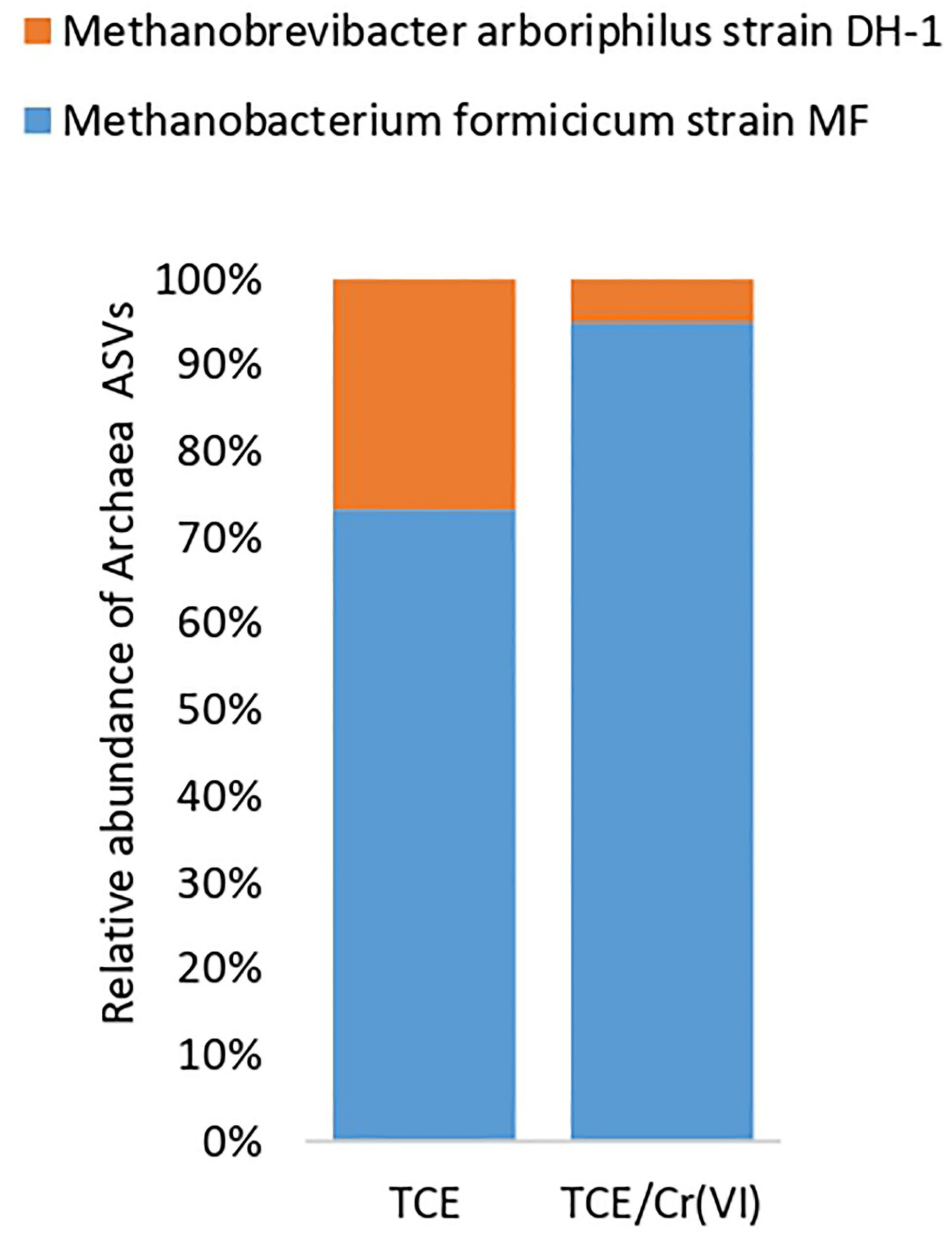
*Archaea* composition of the cathodic biofilm in the reactor operating with TCE and TCE/Cr(VI).

As for the most representative ASVs (Supplementary Table S1) at the genus level (Figure 3B), *D. mccartyi* (ASV1), *Desulfovibrio* (ASV2), *Lentimicrobium* (ASV3), and *Thiobacillus* (ASV6) species were mainly found in the TCE biocathode (Figure 3B). Unidentified species belonging to *Rhodocyclaceae* (ASV4) and *Anaerolineaceae* (ASV5) families were detected among the most abundant ASVs of the TCE biocathode (Figure 3B). The TCE/Cr(VI) biocathode showed a slightly modified microbial composition at the genus level than the reactor’s cathodic biofilm running only with TCE. Higher *D. mccartyi* relative abundance (ASV1) was observed (21.1% on the TCE biocathode; 32.6% on the TCE/Cr(VI) biocathode; Figure 3B). Additionally, *Thermovirga* (ASV14), *Coriobacteria* OPB41 (ASV20), *Brevundimonas* (ASV24, ASV25), *Ochrobactrum* (ASV26), and *Dechloromonas* (ASV27) species were also detected among the most abundant ASVs at genus level on the TCE/Cr(VI) biocathode (Figure 3B). Also, *Desulfovibrio* (ASV2) was found abundant in the system, even if with differences between TCE (17%) and TCE/Cr(VI) (2.4%) biocathode (Figure 3B). Interestingly, on the TCE/Cr(VI) biocathode, some known chromium-resistant bacteria, including *Ochrobactrum* (ASV26, 3%), *Brevundimonas* (ASV24 and ASV25, 5.71%), and *Dechloromonas* (ASV27, 3.17%), were found. Moreover, in line with CH_4_ formation performance (Table 1), the hydrogenotrophic methanogenic consortium with two specialized methanogens, *Methanobrevibacter arboriphilus* strain DH-1 and *Methanobacterium formicicum* strain MF, was identified both in TCE and TCE/Cr(VI) biocathodes by archaeal 16SrRNA gene amplicon sequencing (Figure 5).

### Metagenome Classification, Sequencing, and Assembly Statistics of the TCE/Cr(VI) Biocathode

The metagenomic analysis has been conducted to gain insights into the metabolic features of the leading microbial players occurring at the TCE/Cr(VI) biocathode. A total of 2,110,164 raw DNA sequences were obtained. After the base quality and PhiX-filtered, a total of 2,071,082 trimmed reads before *de novo* metagenome assembly were generated (BioProject: PRJNA670625). The total assembly length of the TCE/Cr(VI) biocathode metagenome was 44.3Mb. The TCR/Cr(VI) metagenome was well assembled and contained few taxa (Supplementary Figure S1A). Four genome bins were extracted based on differential abundance using a kmer-based tSNE approach (t-distributed stochastic neighbor embedding; Supplementary Figure S1B). The extracted genome bins included *D. mccartyi* (Bin 1), *M. formicicum* (Bin 2), *Aeromicrobium* sp. (Bin 3), and *M. arboriphilus* (Bin 4). General characteristics of the four genome bins extracted from the TCE/Cr(VI) biocathode are reported in Supplementary Tables S2 and S3. The classification against the Global Taxonomy Database (GTDB) was subsequently used to infer taxonomy for the extracted genome bins, which were nearly complete (95–100%) and contained low contamination levels (0–1.8%). In the annotated genomes, the identified cluster of orthologous groups of proteins (COGs) mostly fell within functional categories linked to metabolism, information storage and processing, cellular process, and signaling (Supplementary Table S4; Supplementary Figure S2). Within the annotated coding sequences (CDSs), enzymes related to transferases, hydrolases, and oxidoreductases were the most abundant (Supplementary Figure S3).

## Discussion

### Overview of the Microbial Player Involved in the Bioprocesses Occurring in the Bioelectrochemical System

The bioelectrochemical reactor investigated in this study allowed a complete TCE removal *via* RD. Kinetic data demonstrated that the addition of Cr(VI) in the system did not negatively affect the performance of the bioelectrochemical reactor. *Dehalococcoides mccartyi* was found on the biocathode of the system operating both with TCE and TCE/Cr(VI), further demonstrating that the addition of Cr(VI) as a co-contaminant did not negatively affect the RD rate nor the growth of *D. mccartyi*. As reported in Figure 2, the abundance of *D. mccartyi* estimated in the run with TCE/Cr(VI) was higher compared to the initial run with only TCE. This finding was likely due to the establishment of the optimum growth conditions and kinetic performance after about 200days of operating conditions of the bioelectrochemical system (Figure 2).

The Coulombic efficiencies estimated in the reactor evidenced electron consuming mechanisms other than RD, including Cr(VI) to Cr(III) reduction and bioelectromethanogenesis for CO_2_ reduction into CH_4_. The microbiome characterization, including the metagenomic analysis, revealed that a specialized microbial community led the processes occurring in the bioelectrochemical system.

*Dehalococcoides mccartyi* was the most abundant microorganism found in the microbiome of the biocathode of the reactor operating with TCE and TCE/Cr(VI). Additionally, *Desulfovibrio* species were found. They are metabolically versatile microorganisms capable of anaerobic sulfate reduction and RD (Men et al., 2013; Mao et al., 2017; Wen et al., 2017; Lim et al., 2018), already detected in TCE-consortia enriched on a variety of electron donors (Kotik et al., 2013). Some *Desulfovibrio* species (i.e., *D. vulgaris* and *D. desulfuricans*) were previously described as capable of heavy metal reduction in anaerobic environments (Franco et al., 2018). Nevertheless, *Desulfovibrio* ASVs found in our study, including ASV2, ASV7, ASV8, ASV15, and ASV16 (Supplementary Table S1), are phylogenetically affiliated to *Desulfovibrio sulfodismutans* strain ThAcO1 (GenBank: NR_026480.1; Blast alignment: ≥95% similarity), a heterotrophic microorganism capable of growth *via* sulfur disproportionation. It can also grow *via* sulfate reduction coupled to the oxidation of small organic compounds, although slower than disproportionation (Ward et al., 2020). Previous studies highlighted the involvement of *Desulfovibrio* spp. in H_2_ production by direct electron uptake from the biocathode (Carepo et al., 2002; Perona-Vico et al., 2020), suggesting that a similar mechanism may occur in the bioelectrochemical system here described. The latter would be in line with the CE-excess percentage estimated in the system (Table 1).

In addition, on the TCE/Cr(VI) biocathode, some sequences affiliated with known chromium-resistant bacteria were found, corroborating the hypothesis that Cr(VI) reduction in the reactor was probably the result of the combination of both the applied reducing potential and microbial activity. In particular, *Ochrobactrum*, *Brevundimonas*, and *Dechloromonas* species were found. Among them, ASV26 (Supplementary Table S1) was affiliated with *Ochrobactrum anthropic*, a chromium-resistant microorganism isolated from Cr(VI) contaminated environments with Cr(VI) reduction potential being a carrier of chromate resistance genes. *Brevundimonas* ASV24 and ASV25 showed ≥99% similarity to *Brevundimonas diminuta*, a microorganism isolated from a magnetite mine drainage sample with solid tolerance to Cr(VI) (Lu et al., 2011). Additionally, the *Dechloromonas* genus is known for its ability to remove multiple inorganic contaminants, including perchlorate, nitrate, and selenium (Upadhyaya et al., 2019), and was already found as a dominant microorganism in anaerobic Cr(VI)-removing reactors (Chung et al., 2006). Although the analysis showed microorganisms with tolerance and resistance capabilities to Cr(VI), known bacteria directly involved in Cr(VI) bioreduction processes were not detected.

Further, *M. arboriphilus* strain DH-1 and *M. formicicum* strain MF colonized the biocathode. *Methanobrevibacter arboriphilus* strain DH1 (order *Methanobacteriales*) is an autotrophic methanogen isolated from the wetwood of methane-emitting trees (Enzmann et al., 2018), known to grow hydrogenotrophically, utilizing CO_2_ and H_2_ for CH_4_ production (Zeikus and Henning, 1975). Similarly, *M. formicicum* is a hydrogenotrophic methanogen (Bryant and Boone, 1987), and it has been previously reported with *M. arboriphilus* in a bioelectrochemical methanogenic reactor (Sasaki et al., 2013). Interestingly, previous studies evidenced that together with the reduction of CO_2_ to CH_4_ by H_2_ oxidation, Cr(VI) reduction might also be led by methanogens in the presence of specialized electron transfer complex, especially when H_2_ (or direct electrons) are not limiting in the environment (Viti et al., 2014).

Overall, gene amplicon sequencing provided an overview of the microbial player involved in the bioprocesses (i.e., RD, hydrogenotrophic methanogenesis, and Cr(VI) reduction) occurring in the bioelectrochemical system here reported. *D. mccartyi* led TCE-dechlorination. In contrast, *Desulfovibrio* sp. may be involved in bioelectrochemical H_2_ production. Further, *M. arboriphilus* and *M. formicicum* are primarily engaged in hydrogenotrophic CH_4_ production and might play a role in Cr(VI) reduction.

### Metabolic Features of *D. mccartyi* and Methanogens in the TCE/Cr(VI) Biocathode

Metagenomic analysis conducted on the biofilm growing on the TCE/Cr(VI) biocathode reported organochloride respiration and hydrogenotrophic methanogenesis as the driving processes occurring in the bioelectrochemical system. A selected microbial consortium established on the biocathode was highlighted and included the most relevant dechlorinating microorganism *D. mccartyi* and the hydrogenotrophic methanogens *M. arboriphilus* and *M. formicicum*. In line with the 16S rRNA gene amplicon sequencing data, metagenomic investigations did not provide evidence for the presence of the known chromium reducing bacteria directly involved in the Cr(VI) to Cr(III) reduction, strengthening the hypothesis that hydrogenotrophic methanogens may be responsible for Cr(VI) reduction at the biocathode. Several studies conducted on the metabolic pathways of interest (i.e., cobalamin synthesis, methionine synthesis, oxygen scavenging, hydrogen production, and electron donor metabolism) of stable dechlorinating mixed cultures (i.e., dechlorinating cultures: KB1, ANAS, DonnaII) demonstrated that the presence of non-dechlorinating microorganisms, including methanogens, is essential to sustain anaerobic RD processes and *D. mccartyi* growth (Men et al., 2013). In particular, methanogens are crucial for *D. mccartyi* functionality to provide essential metabolites, including corrinoids, and often act as oxygen scavengers. Indeed, despite that methanogens have been often considered hydrogen competitors, several pieces of evidence suggested that this assumption may not be applicable in particular when electrons are exceeded (Hug et al., 2012). This is the case of the bioelectrochemical reactor here reported where unrecovered reducing power accounted for 53.9% in the TCE/Cr(VI) system (Table 1). It is conceivable that interactions instead of competitions were established between *D. mccartyi* and hydrogenotrophic methanogens based on kinetic and metagenomic data. We screened the annotated genomes extracted from TCE/Cr(VI) biocathode, mainly focusing on the primary metabolic features, including RDases as fundamental for the RD process, corrinoid compounds as an essential cofactor for the corresponding RDases, and on the hydrogenases as crucial enzymes for H_2_ metabolism in *D. mccartyi*. The analysis included also the annotated genomes of *M. formicicum* and *M. arboriphilus* as H_2_ consumers for CH_4_ production, cofactor suppliers to *D. mccartyi* for cobalamin biosynthesis, and Cr(VI) reducers.

#### RDases of *D. mccartyi* From TCE/Cr(VI) Biocathode

Among the 1,471 CDSs with proteins of the TCE/Cr(VI) biocathode *D. mccartyi* extracted genome, a total of 29 dehalogenase genes have been annotated (Table 2), including 13 reductive dehalogenase genes (*rdhA*) predicted to encode the catalytically active enzyme (RdhA), one *rdhB* gene encoding the membrane anchor protein, additional 14 genes coding for dehalogenase hypothetical proteins, and one gene encoding a haloacid dehalogenase (GenPept: MBF4482892).

**Table 2:** Reductive dehalogenase genes annotated in the genome of *D. mccartyi* extracted from the TCE/Cr(VI) biocathode.

GenPept accession number	Identity (%)	Similar reductive dehalogenases	*D. mccartyi* strain	Ortholog group	Characterized function
MBF4481786	99.60	DET152	195	33	None
MBF4481792	98.20	DET1528	195	75	None
MBF4481797	98.40	DET1535	195	34	None
MBF4482477	99.80	DET1545	195	15	Expressed during starvation
MBF4482483	99.40	DET1538	195	17	None
MBF4481809	99.80	CG4_X793_RS06795	CG4	10	None
MBF4481838	94.50	CG4_X793_RS06825	CG4	17	None
MBF4482084	99.20	MB_rdhA5	MB	nd	None
MBF4481750	96.20	8658308VS^*^	*VS*	19	None
MBF4481754	95.90	8658312VS^*^	*VS*	40	None
MBF4481741	99.60	DEHALATV1_RS06695^**^	UCH-ATV1	76	None
MBF4481742	96.20	DEHALATV1_RS06700^**^	UCH-ATV1	81	None
MBF4482271 (RdhA)	99.64	Dm11a5_1352^***^	11a5	5	TceA – catalytic subunit
	98.38	KB1338_1	KB-1 consortium	5	TceA – catalytic subunit
	96.57	DET0079_tceA	195	5	TceA – catalytic subunit
MBF4482272	100	rdhB	*Multispecies*	-	reductive dehalogenase membrane-anchoring subunit RdhB
Other hypothetical dehalogenases, uncharacterized:					
GenPept accession number	MBF4481743, MBF4481749, MBF4481753, MBF4481785, MBF4481791, MBF4481796, MBF4481808, MBF4482083, MBF4482154, MBF4482216, MBF4482478, MBF4482484, MBF4481814, and MBF4481844				

* Genes found embedded in distinct genomic islands (GEIs) with different predicted integration sites, suggesting that these genes were acquired horizontally and independently by distinct mechanisms (McMurdie et al., 2009).

** Genes found in *Dehalococcoides* sp. genome where genomic rearrangement occurred during culture (Yohda et al., 2017).

*** Gene from *D. mccartyi* strain 11a5 where a circular extrachromosomal genetic element and a new tetrachloroethene reductive dehalogenase gene were found (Zhao et al., 2017).

The annotated dehalogenases have been run against the Reductive Dehalogenase Database^2^ recently released and based on the orthologous sequence similarity (i.e., Ortholog Groups, OGs; Hug et al., 2013; Molenda et al., 2020). Remarkably, none of the putative reductive dehalogenases encoding proteins shared 100% identity with those identified in the database (Table 2). The *rdhB* gene encoding the membrane anchor protein shared 100% similarity to the TCE reductive dehalogenase membrane-anchoring subunit RdhB (NCBI Reference Sequence: WP_010935885.1), already found in several *D. mccartyi* species (Table 2). Diversely, most of the *rdhA* genes found encoding the catalytically active enzyme showed uncharacterized functions, except for the MBF4482477 RdhA with 99.8% of similarity to DET1545 – highly expressed during starvation in *D. mccartyi* 195 – and the MBF4482271 RdhA with similarity ≥98% to the genes KB1338_1 (from KB-1 consortium) and Dm11a5_1352 (*D. mccartyi* strain 11a5) that corresponded to the TceA catalytic subunit (Table 2). Interestingly, this protein showed only 96.57% of similarity to the gene DET0079_tceA of *D. mccartyi* 195. Among the RdhA found, five shared high similarities (from 98.2 to 99.6%) to known enzymes already detected in *D. mccartyi* 195, including DET152, DET1528, DET1535, DET1545, and DET1538 (Table 2). We also found RdhAses (GenPept: MBF4481741, MBF4481742, MBF4481741, MBF4481742, MBF4482271) similar to those already found in *D. mccartyi* strains *VS*, UCH-ATV1 and 11a5 (8658308VS, 658312VS, DEHALATV1_RS06695, DEHALATV1_RS06700, Dm11a5_1352). The *rdhA* genes of these strains were found embedded in distinct genomic islands with different predicted integration sites (i.e., 8658308VS, 8658312VS; McMurdie et al., 2009), or reported in genomes where genomic rearrangement occurred during culturing (i.e., DEHALATV1_RS06695, DEHALATV1_RS06700; Yohda et al., 2017) or found in *D. mccartyi* strain (11a5) with circular extrachromosomal genetic elements allowing gene mobilization/horizontal transfer (Zhao et al., 2017). Overall, in these strains, gene mobilization occurs. The reductive dehalogenase genes mobilization may occur by distinct mechanisms, and recent studies showed that CRISPR-Cas (Clustered Regularly Interspaced Short Palindromic Repeats – CRISPR associated) system might play a role in *D. mccartyi* genes transfer (Molenda et al., 2019). Interestingly, *D. mccartyi* from TCE/Cr(VI) biocathode is a CRISPR-containing genome (Supplementary Table S3) harboring one CRISPR array with genes coding for the CRISPR-Cas system (Table 3). CRISPR is an adaptive immune system constituted by one or more CRISPR arrays (i.e., AT-rich leader sequence followed by short repeats of 21–48bp that are separated by unique spacers of 26–76bp, homologous to sequences in mobile genetic elements) and several CRISPR-associated (*cas*) genes: When “invading” genetic elements are identified in the genome, CRISPR-Cas system interferes by adding these elements to the CRISPR array.

**Table 3:** Genes linked to the CRISPR-system found in *D. mccartyi* from the TCE/Cr(VI) biocathode.

GenPept accession number	Identity (%)	RefSeq selected product	Description	*D. mccartyi* strain
MBF4481735	98.45	WP_046961576.1	CRISPR-associated helicase/endonuclease Cas3	UCH007 and CG3
MBF4481736	45.18	WP_046961409.1	type I-E CRISPR-associated protein Cse1/CasA	UCH007 and CG3
MBF4481737	88.68	WP_012984524.1	type I-E CRISPR-associated endoribonuclease Cas2	CBDB1, GT, DCMB5, 11a5, KBVC1, SG1, JNA, RC, KS, and GT
MBF4482064	96.65	WP_046961576.1	CRISPR-associated helicase Cas3	UCH007 and CG3
MBF4482400	100	WP_012882749.1	LexA family transcriptional regulator	Multistrain

Till now, only five out of 24 total *D. mccartyi* genomes sequenced so far have been reported to contain CRISPR-Cas genes (strains GT, CBDB1, DCMB5, KBVC1, and KBDCA3), mostly coding for the Class I type I-E system (Huo et al., 2014; Makarova et al., 2015), involved in facilitating or blocking the lateral transfer of *rdhAB* genes among *D. mccartyi* strains. To date, only Molenda et al. (2019) investigated *D. mccartyi* CRISPR-Cas systems, demonstrating that phages and integrative or mobile elements (IMEs) are the most common targets through a site-specific integration in the DNA and that transcriptional regulator (i.e., XRE, LexA, or Cro/IC family) and translocases (i.e., Ftsk/SpoIIIE domain) are involved in this DNA modification system. The analysis of the CRISPR-Cas systems across a growing set of *Dehalococcoides* genomes enabled the discovery of different types of actively replicating extrachromosomal elements targeted by CRISPR-Cas and associated with the mobilization of *rdh* genes that are often located in genomic islands (GIs) and appear to have been acquired horizontally (i.e., *vcrAB* operon, *bvcA* gene; Molenda et al., 2019). These findings explain the common occurrence of a higher *rdhA/*16S rRNA gene copies ratio, mostly found in DNA samples from environmental samples (Molenda et al., 2019). In the annotated *D. mccartyi* genome from the TCE/Cr(VI) biocathode, genes linked to CRISPR-associated helicase/endonuclease (GenPept: MBF4481735), type I-E CRISPR-associated protein Cse1/CasA (GenPept: MBF4481736), type I-E CRISPR-associated endoribonuclease Cas2 (GenPept: MBF4481737) and to CRISPR-associated helicase Cas3 (GenPept: MBF4482064) were found (Table 3). They shared only low similarity (from 45.18 to 98.45%) to CRISPR-associated proteins previously characterized in other *D. mccartyi* strains. The only regulator gene of the CRISPR-Cas system found in *D. mccartyi* from the TCE/Cr(VI) biocathode is the LexA family transcriptional regulator (GenPept: MBF4482400), already present in the majority of *D. mccartyi* strains. These findings, together with the occurrence of *rdhA* genes with high similarity to those already found in strains where *rdhA* mobilization and/or horizontal transfers were reported, suggest that also the genome of *D. mccartyi* extracted from the TCE/Cr(VI) biocathode may harbor metabolic features linked to gene mobilization. However, additional genome walking, including the searching for extrachromosomal DNA, and comparative genomic studies are necessary to gain more insights into this important metabolic feature of the *D. mccartyi* strain extracted from the biocathode here described.

#### Corrinoid Synthesis in *D. mccartyi* and Cofactors Supply From *Methanobacterium formicicum* and *Methanobrevibacter arboriphilus*

**Figure 6:**
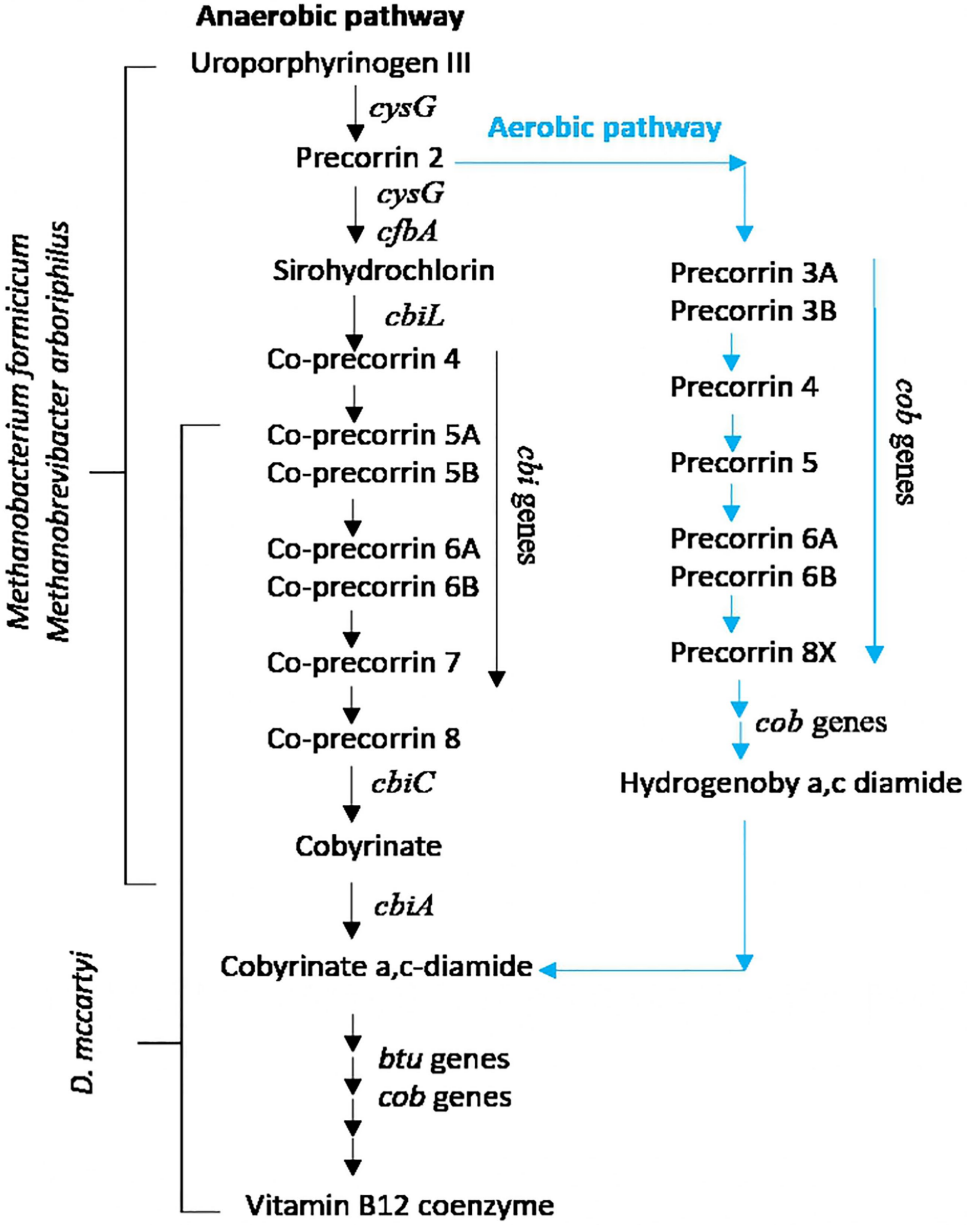
Upstream (KEGG: M00924) and downstream (KEGG: M00122) corrinoid biosynthesis pathways of vitamin B12 coenzyme.

Cobalamins, including vitamin B12, are corrinoid-based essential cofactors for the activity of RDases (Yan et al., 2016). Indeed, RDases are iron–sulfur clusters and cobalamin-containing membrane-bound components of the electron transfer chain that catalyze the H_2_-dependent dechlorination of the substrates (Seshadri et al., 2005). Nevertheless, it is well known that *D. mccartyi* is incapable of *de novo* cobalamin biosynthesis, and all strains harbor only genes involved in the downstream corrinoid biosynthesis pathway, from cobyrinate *a*, *c* diamide to vitamin B12 coenzyme (KEGG pathway: M00122). Thus, *D. mccartyi* is a corrinoid auxotroph microorganism that needs cobamide-producing microbes to supply the required corrinoid cofactors: This implies that the co-presence of non-dechlorinating members (mostly fermentative and/or acetogenic bacteria and/or methanogens) is essential for *D. mccartyi* growth (Yan et al., 2016; Yang et al., 2017). This feature makes it capable of scavenging metabolites (i.e., cofactors) from other microorganisms present in the same environment, such as methanogenic *Archaea*, and transport them into the cell. Metagenomics and kinetic performances suggest that in the TCE/Cr(VI) biocathode, RD activity driven by *D. mccartyi* was also sustained by corrinoid-producing hydrogenotrophic methanogens *M. arboriphilus* and *M. formicicum*. They can perform the upstream corrin ring biosynthesis from uroporphyrinogen III to sirohydrochlorin and finally to cobyrinate a,c-diamide (KEGG pathway: M00924), a precursor of the vitamin B12 coenzyme. The simplified pathway for the upstream (KEGG: M00924) and downstream (KEGG: M00122) corrinoid biosynthesis pathways of vitamin B12 coenzyme is reported in Figure 6.

*Methanobrevibacter arboriphilus* and *M. formicicum* genomes extracted from the biocathode harbor all the anaerobic cobalamin’s genes biosynthesis pathway from uroporphyrinogen III to sirohydrochlorin and finally to cobyrinate a,c-diamide (KEGG pathway: M00924), as a precursor of the vitamin B12 coenzyme. The first gene involved in this pathway is *cysG*, followed by genes *cbi* (*cbiC*, *cbiD*, *cbiE*, *cbiG*, *cbiL*, *cbiM*, *cbiQ*, and *cbiT*), *cfbA*, and *cob* (*cobI*, *cobM*, *cobJ*, *cobK*, *cobH*, and *cobB*). Diversely, in the TCE/Cr(VI) biocathode *D. mccartyi* genome, *cob* genes (*cobC*, *cobD*, *cobU*, *cobS*, *cobT*, and *cobN* genes, Table 4) were found.

**Table 4:** Genes involved in the anaerobic cobalamin synthesis pathway in the genome of *D. mccartyi* from TCE/Cr(VI) biocathode.

GenPept accession number	Gene	Function
MBF4482113	*cbiE*	Precorrin-6y C5,15-methyltransferase (decarboxylating) subunit CbiE
MBF4481778	*cbiD*	Cobalt-precorrin-5B (C(1))-methyltransferase
MBF4481765	*cobH*	Precorrin-8X methylmutase
MBF4481776	*cobI*	Precorrin-2 C(20)-methyltransferase
MBF4481774	*cobJ*	Precorrin-3B C(17)-methyltransferase
MBF4481775	*cobM*	Precorrin-4 C(11)-methyltransferase
MBF4482814	*cobC*	Alpha-ribazole phosphatase [*Dehalococcoides mccartyi*]
MBF4482117	*cobD*	Cobalamin biosynthesis protein CobD
MBF4482815	*cobU*	Bifunctional adenosylcobinamide kinase/adenosylcobinamide-phosphate guanylyltransferase
MBF4482813	*cobS*	Adenosylcobinamide-GDP ribazoletransferas
MBF4482812	*cobT*	Nicotinate-nucleotide--dimethylbenzimidazole phosphoribosyltransferase
MBF4481784	*cobN*	Cobaltochelatase subunit CobN
MBF4482806	*btuF*	Vitamin B12 ABC transporter, B12-binding component BtuF

These genes are involved in the lower part of the cobalamin biosynthesis from cob(II)yrinate a,c, diamide to Vitamin B12 coenzyme (KEGG).

They typically encode proteins involved in the anaerobic cobalamin synthesis (KEGG pathway: M00122; Yan et al., 2016). *cbiD* and *cbiE* genes were also found: They are involved in the KEGG pathway M00924, from co-precorrin 5B to co-precorrin-6A and from co-precorrin-7 to co-precorrin-8 (Figure 6), and are present in some *D. mccartyi* strains (IBARAKI, DCMB5, CBDB, MB, FL2, 11a5, and CG1/4/5) and ANAS consortium (Brisson et al., 2012). Remarkably, we also found *cobH*, *cobI*, *cobJ*, and *cobM* genes (Table 4). These genes are typical of the upstream aerobic corrin ring synthesis pathway (KEGG pathway: M00925) from precorrin-2 to cobyrinate a,c-diamide. Nevertheless, a recent study showed that *cbi* genes in the anaerobic pathway are orthologous to *cob* genes in the aerobic pathway and are not exclusive to genomes with the aerobic or anaerobic pathways (Shelton et al., 2019). Therefore, the authors suggested that aerobic or anaerobic corrin ring biosynthesis pathways cannot be distinguished based on their annotated gene content, presumably because portions of the two pathways share orthologous genes. Additionally, B12-binding component BtuF of the vitamin B12 transporter has been found in the *D. mccartyi* genome from the TCE/Cr(VI) biocathode. Our findings suggest that in the TCE/Cr(VI) biocathode, *M. formicicum* and *M. arboriphilus* play a role in *D. mccartyi* growth by sustaining the complete cobalamin synthesis pathway (Figure 6).

#### H_2_ Metabolism

*Dehalococcoides mccartyi* can exclusively use H_2_ as an electron donor for the anaerobic dechlorination making H_2_ metabolism fundamental for its physiology. *Dehalococcoides mccartyi* commonly harbors genes encoding five different hydrogenase complexes such as cytoplasmic (Vhu) and four membrane-bound (Hup, Hyc, Ech, and Hym) hydrogenases (Kube et al., 2005; Seshadri et al., 2005; Morris et al., 2007; Schipp et al., 2013). Most of them are [NiFe]-hydrogenases, while the H_2_-uptake (Hup) hydrogenase is a [Fe]-hydrogenase, the main enzyme involved in H_2_-driven organohalide respiration (Seshadri et al., 2005). The Hup enzyme involves a direct transfer of the electrons derived from H_2_ oxidation *via* protein–protein interactions, including ferredoxin-like proteins, which resemble electron-transferring subunits of oxidoreductases (Kublik et al., 2016; Hartwig et al., 2017; Seidel et al., 2018). In line with the general metabolic features of *D. mccartyi*, in the genome extracted from the TCE/Cr(VI) biocathode, hydrogenase complexes Hyp, Hyc, Hym, Vhu, and Ech were found (Table 5).

**Table 5:** Genes encoding hydrogenases in *D. mccartyi* from TCE/Cr(VI) biocathode.

GenPept accession number	Gene	Function
MBF4481889	hypA	Hydrogenase maturation nickel metallochaperone HypA
MBF4481888	hypB	Hydrogenase nickel incorporation protein HypB
MBF4481885	hypD	Hydrogenase formation protein HypD
MBF4481884	hypE	Hydrogenase expression/formation protein HypE
MBF4481887	hypF	Hydrogenase maturation factor HypF
MBF4483122	hyaD/hybD	HyaD/HybD family hydrogenase maturation endopeptidase
MBF4482458	hycC	Hydrogenase membrane subunit
MBF4481886	hypC/hybG/hupF	HypC/HybG/HupF family hydrogenase formation chaperone
MBF4483158	hymD	[Fe] hydrogenase, HymD subunit
MBF4483157	hymC	[Fe] hydrogenase, small subunit
MBF4482774	vhuA	Ni/Fe hydrogenase subunit alpha
MBF4482773	vhuG	Methyl viologen-reducing hydrogenase VhuG
MBF4482967	echE	Ni-dependent hydrogenase large subunit
MBF4482820	cdhC	CO dehydrogenase/CO-methylating acetyl-CoA synthase complex subunit beta
MBF4482137	FrhB/FdhB	Coenzyme F420 hydrogenase/dehydrogenase, beta subunit C-terminal domain
MBF4483124	-	Ni/Fe hydrogenase small subunit hydrogenase
MBF4482460	-	Hydrogenase-4 component E
MBF4482775^*^	-	Hydrogenase maturation protease
MBF4482461^*^	-	Hydrogenase membrane subunit
MBF4483123^*^	-	Ni-dependent hydrogenase large subunit

* No similar proteins were found in other *D. mccartyi* strains.

In detail, genes encoding protein subunits linked to membrane-bound [Ni-Fe]-hydrogenase included *hyc* genes (*hycC/hybG*) encoding the formate hydrogenlyase subunits of the membrane-bound hydrogenase, and *hyp* genes (*hypD*, *hypE*, *hypB*, and *hypD*) encoding the carbamoyl dehydratase (HypE), carbamoyltransferase (HypF) subunits and the hydrogenase maturation factors (HypB and HypD). Additionally, *hyb* genes (*hybB*, *hybD*, and *hybF*) encoding the putative [Ni-Fe]-hydrogenase 2 b-type cytochrome subunit (HybB), the hydrogenase 2 maturation protease (HybD), and the hydrogenase maturation factor (HybF) were detected. *hydB* gene encoding the periplasmic [Ni-Fe]-hydrogenase large subunit and *hyf* genes (*hyfB* and *hyfG*) encoding the hydrogenase-4 components were also present. Vhu and Ech enzymes were found, the latter probably involved in generating the low-potential electrons for biosynthesis rather than being involved with organohalide respiration (Morris et al., 2007). Moreover, the gene *cdhC* encoding the CO dehydrogenase/CO-methylating acetyl-CoA synthase complex (GenPept: MBF4482820) and the genes *frhB*/*fhdB* encoding the coenzyme F420 hydrogenase/dehydrogenase complex (GenPept: MBF4482137) were also present in the TCE/Cr(VI) biocathode *D. mccartyi* genome (Table 5). Additionally, in line with the bioelectromethanogenesis occurring in the system, all the genes involved in CH_4_ formation from H_2_ and CO_2_ (KEGG pathway: M00567) were found in the annotated genomes of *M. arboriphilus* and *M. formicicum* (Table 6).

**Table 6:** Genes involved in the CH_4_ formation pathway from H_2_ and CO_2_ (KEGG: M00567) found in the annotated genome of *Methanobacterium formicicum* (GenBank: JADIIL000000000) and *Methanobrevibacter arboriphilus* (GenBank: JADIIN000000000) from the TCE/Cr(VI) biocathode and the corresponding functions.

Gene	Function
fwdA,B,C,D	Formylmethanofuran dehydrogenase subunits A, C, B, D
fdhD, fdhF	Formate dehydrogenase
mob	Molybdopterin-guanine dinucleotide biosynthesis protein B
ftr, fhcD	Formylmethanofuran--tetrahydromethanopterin N-formyltransferase
mch	Methenyltetrahydromethanopterin cyclohydrolase
mer	5,10-methylenetetrahydromethanopterin reductase
mtd	F420-dependent methylenetetrahydromethanopterin dehydrogenase
mcrA, mcrB, mcrG	Coenzyme-B sulfoethylthiotransferase subunits alpha (A), beta (B), gamma (G)
mcrC, mcrD1, mcrD2	Methyl-coenzyme M reductase I operon protein C, D1, D2
mtrA, mtrC, mtrD, mtrE, mtrF, mtrG, mtrH	Tetrahydromethanopterin S-methyltransferase subunits A, C, D, E, F, G, H
frhA, frhB, frhG, frhD	Coenzyme F420 hydrogenase subunit alpha(A), beta(B), gamma(G); coenzyme F420-reducing hydrogenase FrdhD
mvhA/mvhG	F420-non-reducing hydrogenases
hdrB, hdrC	CoB-CoM heterodisulfide reductase subunits B, C

#### Cr(VI)-to-Cr(III) Bioreduction

Cr(VI) to Cr(III) reduction occurring in the bioelectrochemical system resulted from the combination of both the applied reducing potential and microbial activity. Nevertheless, amplicon sequencing showed only the presence of AVS26 (3%) affiliated with the known Cr(VI) resistant *Ochrobactrum*, and bacteria linked to direct Cr(VI) reduction were not detected. Interestingly, previous studies reported that extracellular Cr(VI) reduction might occur with H_2_ as an electron donor in the presence of hydrogenases and through the action of *c*-type cytochromes and membrane reductase, such as the mtrCAB complex, essential components for extracellular electron transfer (Marsili et al., 2008; von Canstein et al., 2008). Previous studies demonstrated that this Cr(VI) reduction mechanism, together with the reduction of CO_2_ to methane by H_2_ oxidation, might be led by methanogens in the presence of the electron transfer c-cytochrome/mtrCAB complex, especially when H_2_ (or direct electrons) are not limiting in the environment (Viti et al., 2014; Singh et al., 2015). In line with these previous reports, we found that *M. formicicum* genome of the TCE/Cr(VI) biocathode harbors genes encoding c-cytochrome (GenPept: MBF4475800) and mtrCAB complex (GenPept: MBF4473862, MBF4474248, MBF4473860; Table 6). Diversely, *M. arboriphilus* from the TCE/Cr(VI) biocathode harbors genes encoding mtrCAB complex, but no cytochromes have been found in the annotated genome. Additionally, intracellular Cr(VI) reduction mechanisms have been reported in methanogens in the presence of H_2_ as electron donor and with available transport systems for inorganic solutes formed by the ABC family of the ATP-dependent transporters. This system allows Cr(VI) transport into the cells, mainly if moderate Cr(VI) concentrations are present in the medium to avoid the cytotoxic effect. Thus, Cr(III) reduction occurs within the cell by proteins similar to NADPH-dependent FMN reductase in *Methanobacterium* sp. (NCBI accession number: YP_004518865; Smith et al., 1997). Interestingly, in the TCE/Cr(VI) biocathode *M. formicicum* genome, we found 16 genes encoding ABC-transporter ATP-dependent, 17 genes encoding ABC-transporter permeases, and one metal ABC transporter permease in addition to the gene *npdG* encoding the NADPH-dependent F420 reductase (GenPept: MBF4475652). Diversely, in the *M. arboriphilus* genome of the TCE/Cr(VI) biocathode, 11 genes encoding ABC-transporter ATP-dependent and 10 genes encode the ABC-transporter permeases were found (Table 6). At the same time, no NADPH-dependent F420 reductase was present. These findings suggested that *M. formicicum* on the TCE/Cr(VI) biocathode may be responsible for the extracellular and/or intracellular hydrogenotrophic Cr(VI) reduction, together with hydrogenotrophic CH_4_ production.

## Conclusion

**Figure 7:**
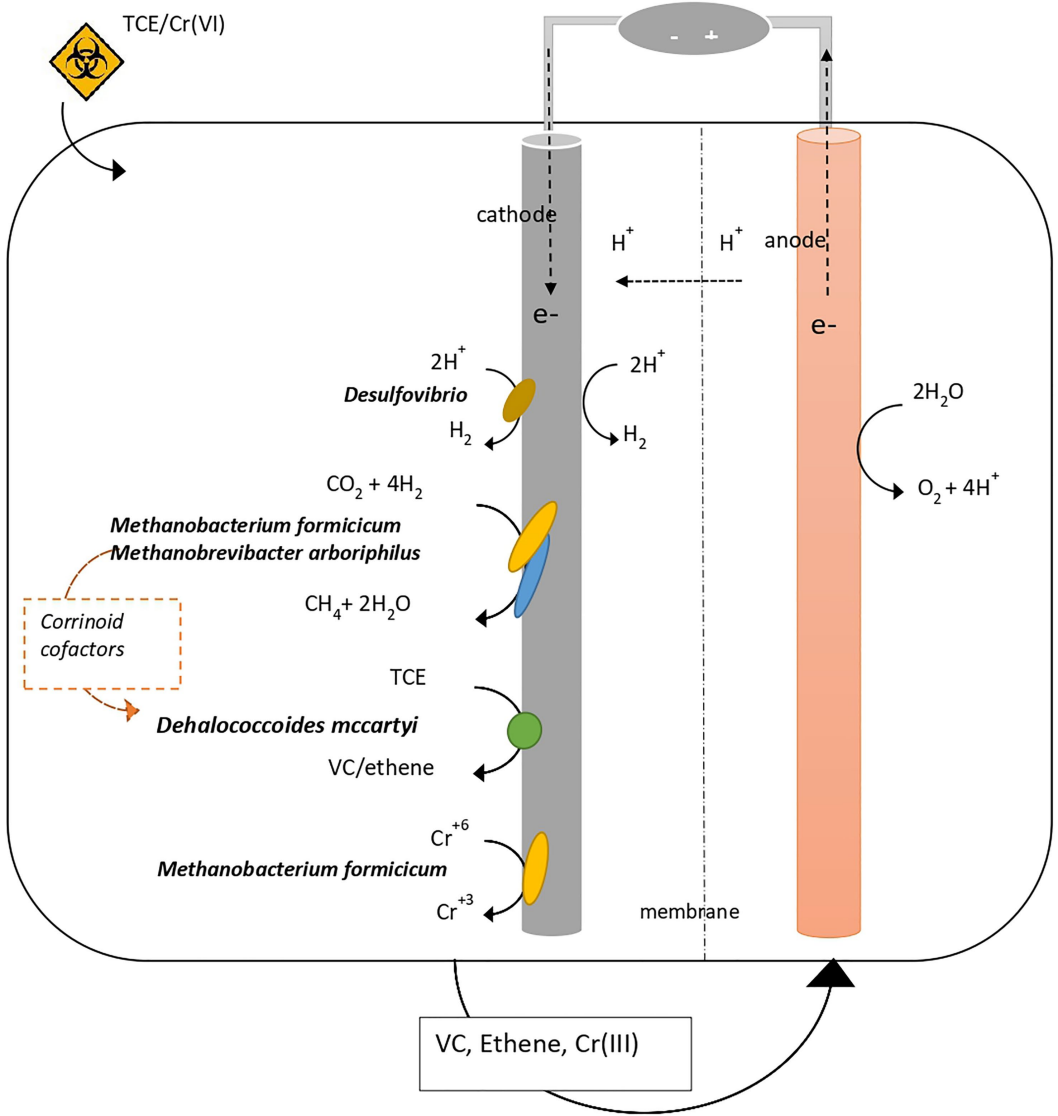
Microbial interactions revealed at the biocathode of the bioelectrochemical system where RD, hydrogenotrophic methanogenesis, and Cr(VI) reduction occur.

The present study reported the microbial and (meta)genomic characterization of a bioelectrochemical system treating TCE- and TCE/Cr(VI)-contaminated water, allowing to elucidate the microbial interactions occurring at the biofilm growing on the cathode (Figure 7). The main processes occurring in the system were RD, hydrogenotrophic methanogenesis, and Cr(VI) reduction. The genomic and metagenomic analysis of the microbial community growing at the biocathode allowed the identification of the key-microbial players: *D. mccartyi* as the unique OHRB, *Desulfovibrio* as H_2_ producer, *M. formicicum*, and *M. arboriphilus* as H_2_ consumers for CH_4_ production, cofactor suppliers for cobalamin biosynthesis and Cr(VI) reducers. *D. mccartyi* genome extracted from the TCE/Cr(VI) biocathode showed high similarity with *D. mccartyi 195* even if it harbors several uncharacterized RdhAse, including some similar to those found in *D. mccartyi strains* with IMEs (*VS*, *UCH-ATV1*, and *11a5*), and genes involved in the CRISPR-Cas system. These findings suggest that the genome of *D. mccartyi* extracted from the TCE/Cr(VI) biocathode may harbor metabolic features linked to *rdhA* gene mobilization. Further genomic and/or transcriptomic evaluation is necessary to gain more insights into the gene-transfer capabilities. Concerning the H_2_ uptake, *D. mccartyi* genome analysis confirmed this as the only electron supply mechanism. As the genome lacks systems for the direct electron transfer into the cell (i.e., cytochromes), most likely electroactivity is not a property of *D. mccartyi*. Pure culture studies are necessary to fulfil this hypothesis.

The role of hydrogenotrophic methanogens *M. formicicum* and *M. arboriphilus* in supporting the growth of *D. mccartyi* has been here highlighted. Indeed, the presence of a complete cobalamin synthesis pathway represents an essential supply of corrinoid-based crucial cofactors for the activity of RDases in *D. mccartyi*. Further, evidence of Cr(VI) to Cr(III) reduction mediated by *M. formicicum* in the TCE/Cr(VI) biocathode has been reported. Indeed, optimal conditions (i.e., H_2_ availability and moderate Cr(VI) concentration) and the presence of c-cytochrome/mtrCAB complex in the annotated genome suggested that *M. formicicum* harbors the metabolic feature for extracellular Cr(VI) reduction. Additionally, the metabolic potential for intracellular Cr(VI) reduction in *M. formicicum* is also sustained by the presence in the genome of genes encoding transport systems for inorganic solutes, and NADPH-dependent reductases, whose involvement in Cr(VI) reduction has been already reported in Cr(VI)-reducing bacteria (i.e., *Shewanella* sp.).

## Data Availability Statement

The datasets presented in this study can be found in online repositories. The names of the repository/repositories and accession number(s) can be found at: https://www.ncbi.nlm.nih.gov/, JADIIK000000000-JADIIN000000000.

## Author Contributions

BM: conceptualization, methodology, data curation, and writing – original draft preparation. MZ: conceptualization, investigation, methodology, and writing – original draft preparation. AL: conceptualization, investigation, and methodology. MM and SR: conceptualization, writing – review and editing, supervision, and funding acquisition. All authors contributed to the article and approved the submitted version.

## Funding

This study was supported by the European Union’s Horizon 2020 Project ELECTRA (www.electra.site) under grant agreement no. 826244.

## Conflict of Interest

The authors declare that the research was conducted in the absence of any commercial or financial relationships that could be construed as a potential conflict of interest.

## Publisher’s Note

All claims expressed in this article are solely those of the authors and do not necessarily represent those of their affiliated organizations, or those of the publisher, the editors and the reviewers. Any product that may be evaluated in this article, or claim that may be made by its manufacturer, is not guaranteed or endorsed by the publisher.

## Footnotes

^1^ https://www.arb-silva.de/documentation/release-132/

^2^ https://rdasedb.biozone.utoronto.ca/
