# Corrigendum: Metagenomic Analysis Reveals Microbial Interactions at the Biocathode of a Bioelectrochemical System Capable of Simultaneous Trichloroethylene and Cr(VI) Reduction

**DOI:** 10.3389/fmicb.2022.879964

**Published:** 2022-03-11

**Authors:** Bruna Matturro, Marco Zeppilli, Agnese Lai, Mauro Majone, Simona Rossetti

**Affiliations:** ^1^Water Research Institute, IRSA-CNR, Rome, Italy; ^2^Department of Chemistry, Sapienza University of Rome, Rome, Italy

**Keywords:** reductive dechlorination, Cr(VI) reduction, bioelectrochemical remediation, *Dehalococcoides mccartyi*, *Methanobacterium formicicum*, *Methanobrevibacter arboriphilus*

An author name was incorrectly spelled as Zepilli. The correct spelling is Zeppilli.

The authors apologize for this error and state that this does not change the scientific conclusions of the article in any way. The original article has been updated.

## Publisher's Note

All claims expressed in this article are solely those of the authors and do not necessarily represent those of their affiliated organizations, or those of the publisher, the editors and the reviewers. Any product that may be evaluated in this article, or claim that may be made by its manufacturer, is not guaranteed or endorsed by the publisher.

