# Supplementary material for: Metagenomic Analysis Reveals Microbial Interactions at the Biocathode of a Bioelectrochemical System Capable of Simultaneous Trichloroethylene and Cr(VI) Reduction
Source: Front Microbiol. 2021 Sep 30;12:747670. doi: 10.3389/fmicb.2021.747670 (PMC8516407; doi:10.3389/fmicb.2021.747670)
Supplement: Supplementary file 1 [file Data_Sheet_1.PDF]

Supplementary Material

Metagenomic analysis reveals microbial interactions at the biocathode of a bioelectrochemical system capable of simultaneous trichloroethylene and Cr(VI) reduction

Bruna Matturro[1*], Marco Zepilli[2], Agnese Lai[2], Mauro Majone[2], Simona Rossetti[1]

[1]*Water Research Institute (IRSA-CNR), Via Salaria km 29.300, 00015 Monterotondo (RM), Italy*
[2]*Department of Chemistry, Sapienza University of Rome, P.le Aldo Moro 5, 00185 Rome, Italy*
**Corresponding author: matturro@irsa.cnr.it*

Table S1. Most abundant ASVs found on the biocathode of the bioelectrochemical system investigated by 16S rRNA gene amplicon sequencing.

ASV ID	ASV SEQUENCE	Phylogenetic affiliation
**ASV1**	85fe11268ae72d0f1f06dc940db825c1	Chloroflexi_Dehalococcoidia_Dehalococcoidales_Dehalococcoidaceae_Dehalococcoides
**ASV2**	c04da21a40b9d3bf8b736f2fe4826d47	Deltaproteobacteria_Desulfovibrionales_Desulfovibrionaceae_Desulfovibrio
**ASV 3**	b2ca34fded61ade07b3bb286aada6fc8	Bacteroidetes_Bacteroidia_Sphingobacteriales_Lentimicrobiaceae_Lentimicrobium
**ASV4**	bf738ab7b8767645cceef6cf6a28af9f	Gammaproteobacteria_Betaproteobacteriales_Rhodocyclaceae
**ASV5**	eddcc3248a0cb51954c114f4ad05a3ee	Chloroflexi_Anaerolineae_Anaerolineales_Anaerolineaceae
**ASV6**	fe5d6129efef0233dc9768f872371d28	Gammaproteobacteria_Betaproteobacteriales_Hydrogenophilaceae_Thiobacillus
**ASV7**	f4687444be39935731655f0145887ea8	Deltaproteobacteria_Desulfovibrionales_Desulfovibrionaceae_Desulfovibrio
**ASV8**	216be078176757dbc545565e09967e03	Deltaproteobacteria_Desulfovibrionales_Desulfovibrionaceae_Desulfovibrio
**ASV9**	b837b9cd2f38ac9a09fc41da38db45ba	Firmicutes_Clostridia_Clostridiales_Family XI_Soehngenia
**ASV10**	75a01961e0f628db181b0af20e5d9684	Firmicutes_Clostridia_Clostridiales_Family XI_Soehngenia
**ASV11**	f49985fedc661ea0adf74b40066e8e7f	Firmicutes_Clostridia_Clostridiales_Family XI_Soehngenia
**ASV12**	bf5f0d3d78f22fc69bc2c1f0d4f50445	Deltaproteobacteria_Desulfuromonadales_Desulfuromonadaceae
**ASV13**	0f8ecdb8910220c38cff0da79399f7bf	Firmicutes_Clostridia_Clostridiales_Family XI_Soehngenia
**ASV14**	9c19dccd11a91cba84100db612f20ec9	Synergistetes_Synergistia_Synergistales_Synergistaceae_Thermovirga
**ASV15**	9a86fd829d0b358daa3f89e9ca8ef00a	Deltaproteobacteria_Desulfovibrionales_Desulfovibrionaceae_Desulfovibrio
**ASV16**	1d482429446fe13dfbec86913fe48d2f	Deltaproteobacteria_Desulfovibrionales_Desulfovibrionaceae_Desulfovibrio
**ASV17**	a597cc514881f238d97c800f30603eb6	Bacteroidetes_Bacteroidia_Bacteroidales_Dysgonomonadaceae_Petrimonas
**ASV18**	d611b8a13a427110419ca8c08435f02d	Synergistetes_Synergistia_Synergistales_Synergistaceae_Thermovirga
**ASV19**	4843600b20db9ac680b75b8a8837140c	Bacteroidetes_Bacteroidia_Bacteroidales_Rikenellaceae
**ASV20**	facb60f9fcf679ac571937fdbb45d80a	Actinobacteria_Coriobacteriia_OPB41
**ASV24**	f12866b5a52589408314b00c2b546060	Alphaproteobacteria_Caulobacterales_Caulobacteraceae_Brevundimonas
**ASV27**	c44a10d45fc73b7caa1d2435738c230a	Gammaproteobacteria_Betaproteobacteriales_Rhodocyclaceae_Dechloromonas
**ASV26**	f5d0d5cbda97f9e460a7662fc8edef58	Alphaproteobacteria_Rhizobiales_Rhizobiaceae_Ochrobactrum
**ASV22**	69c01785f8b99cd83c250b0c4b5056ff	Chloroflexi_Anaerolineae_Anaerolineales_Anaerolineaceae
**ASV25**	d16af5d38041d3681d1dc9d1a6972f99	Alphaproteobacteria_Caulobacterales_Caulobacteraceae_Brevundimonas
	a6572c9de4dcefc3c11cf5a409792e22	Chloroflexi_Dehalococcoidia_Dehalococcoidales_Dehalococcoidaceae_Dehalococcoides (ASV23)
	eb9cdb166ac93e8aec317add385a322a	Bacteroidetes_Bacteroidia_Bacteroidales_Dysgonomonadaceae_Petrimonas (ASV21)

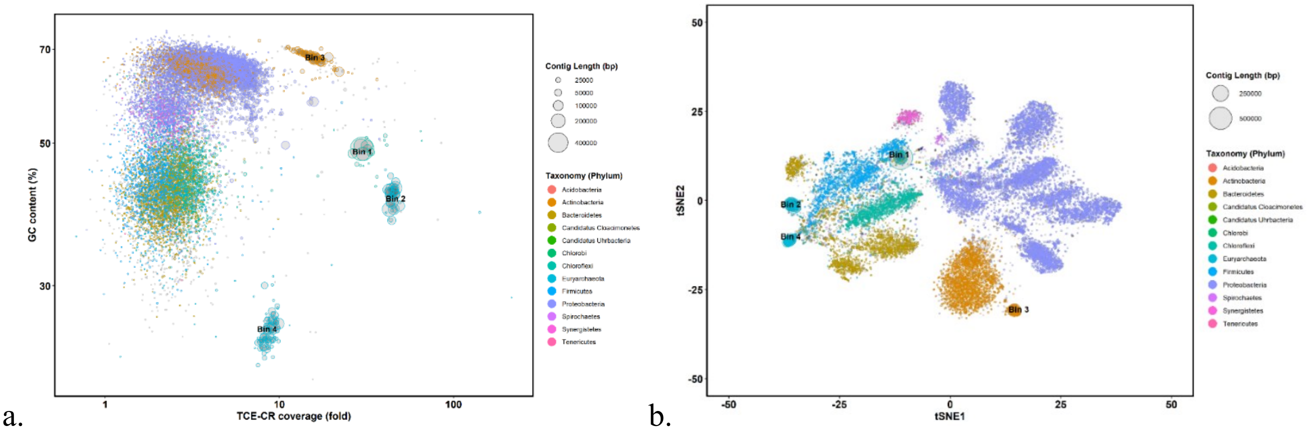

a. b.

Figure S1. TCE/Cr biocathode metagenome. *In plot (a), the x-axis shows the average data coverage (fold), and the y-axis shows the weighted GC-content (percentage of G and C nucleotides). Colors denote phylum-level taxonomic classification, based on the essential genes identified on the contigs. Contigs with no color either contain no essential genes or could not be assigned a phylum level classification. The size of the circles represents the length of the contigs. Plot (b) reports a Kmer-based tSNE (T-distributed Stochastic Neighbor Embedding), highlighting the extracted bins. The size of the circles represents the length of the contigs (bp). Color denotes phylum-level taxonomic classification based on the essential genes identified on the contigs.*

Table S2. Classification, sequencing, and assembly statistics of each bin. Contigs, genome size (bp), GC-nucleotides contents (GC %), genome completeness, and % of contamination of each bin estimated using unique marker genes are reported. N50 = the shortest contig length needed to cover 50 % of the genome was likewise determined.

**Genome bins**	**Genome accession**	**Contigs**	**Genome (N50)**	**Genome size (bp)**	**GC (%)**	**Completeness (%)**	**Contamination (%)**
Bin1: Dehalococcoides mccartyi	JADIIK000000000	23	203441	1437836	49%	99.0	0
Bin 2: Methanobacterium formicicum	JADIIL000000000	40	103973	2442285	41%	99.2	0
Bin 3: Aeromicrobium sp.	JADIIM000000000	60	60655	2072249	68%	94.6	1.74
Bin 4: Methanobrevibacter arboriphilus	JADIIN000000000	83	44031	2347078	26%	100	0.8

Table S3. PGAP gene annotation for each bin extracted from the TCE/Cr(VI) biocathode.

	Methanobrevibacter arboriphilus	Methanobacterium formicicum	Aeromicrobium sp.	Dehalococcoides mccartyi
Genes (total)	2,043	2,469	2,058	1,551
CDSs (total)	2,007	2,420	2,008	1,499
Genes (coding)	1,999	2,402	2,001	1,471
CDSs (with protein)	1,999	2,402	2,001	1,471
Genes (RNA)	36	49	50	52
rRNAs	-	3 (5S)	1, 1, 1 (5S, 16S, 23S)	1, 1, 1 (5S, 16S, 23S)
complete rRNAs	-	1 (5S)	1, 1, 1 (5S, 16S, 23S)	1, 1, 1 (5S, 16S, 23S)
partial rRNAs	-	2 (5S)		
tRNAs	34	44	43	47
ncRNAs	2	2	4	2
Pseudo Genes (total)	8	18	7	28
CDSs (without protein)	8	18	7	28
CRISPR Arrays	-	-	1	1

Table S4. A number of genes within identified Clusters of Orthologous Groups (COGs) falling into the functional categories.

	Dehalococcoides mccartyi (JADIIK000000000)	Aeromicrobium sp. (JADIIM000000000)	Methanobacterium formicicum (JADIIL000000000)	Methanobrevibacter arboriphilus (JADIIN000000000)
**(J) Translation, ribosomal structure, and biogenesis**	81	100	140	141
**(E) Amino acid transport and metabolism**	64	100	61	56
**(C) Energy production and conversion**	53	65	86	69
**(H) Coenzyme transport and metabolism**	48	72	71	59
**(P) Inorganic ion transport and metabolism**	38	41	24	10
**(F) Nucleotide transport and metabolism**	30	37	29	32
**(L) Replication, recombination, and repair**	30	31	33	34
**(R) General function prediction only**	27	28	26	26
**(I) Lipid transport and metabolism**	25	31	18	12
**(T) Signal transduction mechanisms**	25	32	24	12
**(O) Posttranslational modification, protein turnover, chaperones**	20	28	14	14
**(V) Defense mechanisms**	18	21	6	3
**(K) Transcription**	17	34	22	18
**(G) Carbohydrate transport and metabolism**	15	33	22	22
**(M) Cell wall/membrane/envelope biogenesis**	9	48	19	13
**(U) Intracellular trafficking, secretion, and vesicular transport**	7	8	6	5
**(N) Cell Motility**	5	8	0	0
**(S) Function unknown**	5	19	14	14
**(D) Cell cycle control, cell division, chromosome partitioning**	4	13	2	1
**(Q) Secondary metabolites biosynthesis, transport, and catabolism**	3	3	1	0
**(A) RNA processing and modification**	0	0	1	0
**(B) Chromatin structure and dynamics**	0	0	0	0
**(W) Extracellular structures**	0	0	0	0
**(X) Mobilome: prophages, transposons**	0	3	0	0

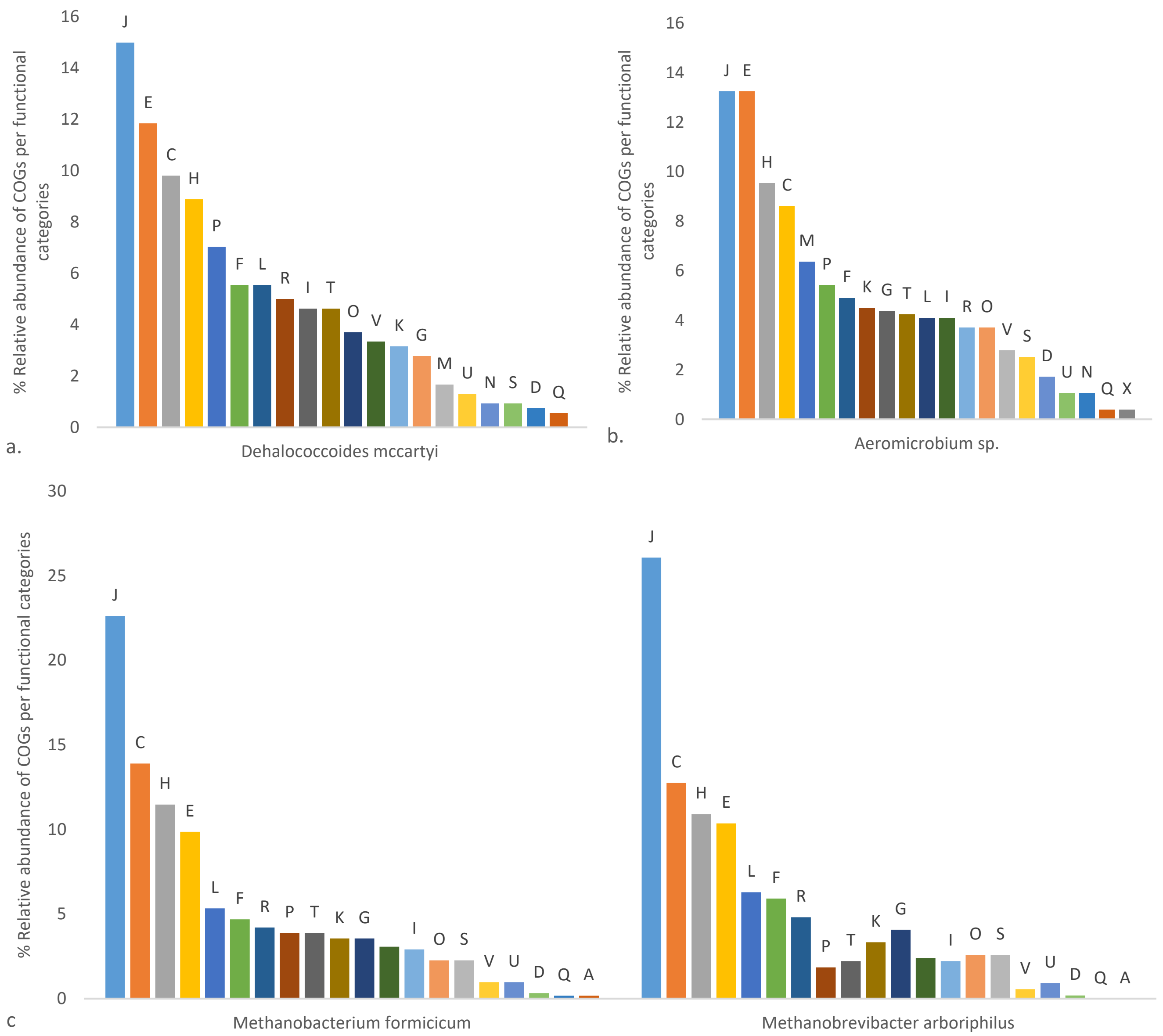

Figure S2. The total numbers of COGs per functional categories are reported for each annotated bin (a. D.mccartyi; b. Aeromicrobium sp.; c. Methanobrevibacterium formicicum; d. Methanobrevibacter arboriphilus) from the whole metagenomic analysis of TCE/Cr(VI) biocathode. One-letter abbreviations for the functional categories: J, translation, including ribosome structure and biogenesis; L, replication, recombination and repair; K, transcription; O, molecular chaperones and related functions; M, cell wall structure and biogenesis and outer membrane; N, secretion, motility and chemotaxis; T, signal transduction; P, inorganic ion transport and metabolism; C, energy production and conversion; G, carbohydrate metabolism and transport; E, amino acid metabolism and transport; F, nucleotide metabolism and transport; H, coenzyme metabolism; I, lipid metabolism; D, cell division and chromosome partitioning; R, general functional prediction only; S, no functional prediction. COGs linked to cellular process and signaling include functional categories: D, N, M, Z, V, W, U, Y, O, T. COGs linked to information storage and processing include functional categories: B, A, L, K, J. COGs linked to metabolism include functional categories: E, G, H, C, P, I, F, Q. Poorly characterized functional categories are S and R.

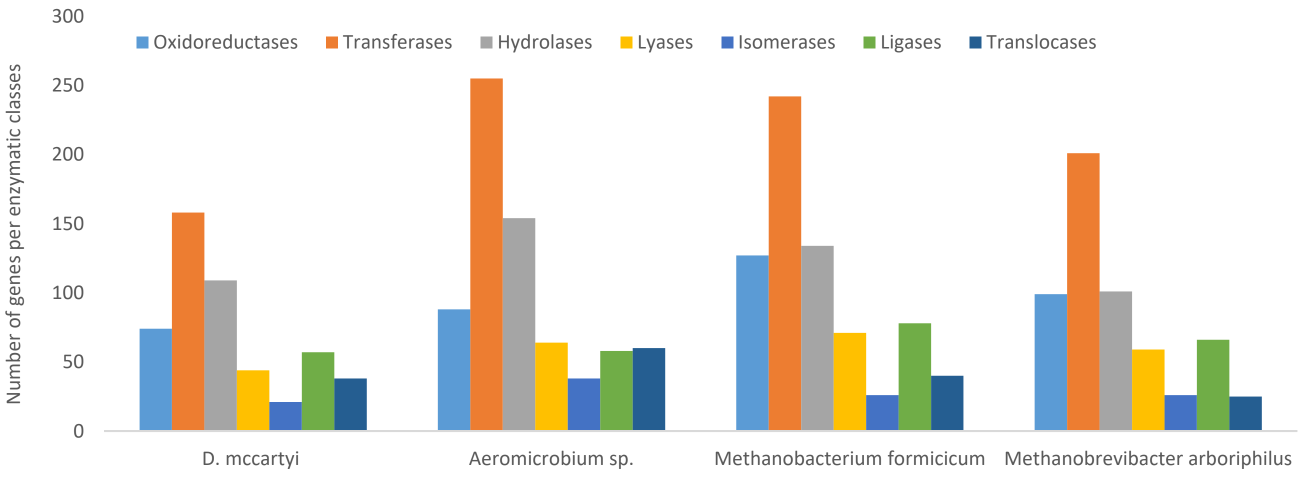

Figure S3. The number of genes annotated for each draft genome falling within oxidoreductases, transferases, hydrolases, lyases, isomerases, ligases, and translocases class enzymes.

General features of annotated genome bins from the TCE/Cr(VI) biocathode

Dehalococcoides mccartyi **(GenBank: JADIIK000000000).** The genome has been assembled in 23 contigs with an N50 of 0.2 Mb. The draft genome is quite complete (99% of completeness and absence of contamination) with a total length of 1.43 Mb and 49% of G+C content (Tab. S2). A comparison of the extracted *D. mccartyi* genome bin (Bin 1) with the *Dehalococcoides mccartyi* 195 (NCBI Reference Sequence: NC_002936.3) and a *D. mccartyi* strain from an actively PCE-to-ethene dechlorinating culture containing ≥ 80% of *D. mccartyi* (GenBank: JAEMVJ000000000), returned average nucleotide identities of 97.64 % and 99.93 %, respectively. A total of 1499 predicted coding DNA sequences (CDSs) were annotated including 1471 CDSs with proteins (Tab. S3). Identified COGs mostly fell within functional categories linked to metabolism (52%), information storage and processing (24.2%), and cellular process and signaling (16.8%) (Tab. S4, Fig. S2). Within the annotated CDSs, those related to transferases, hydrolases, and oxidoreductases were the most abundant (Fig. S3).

Methanobacterium formicicum **(GenBank: JADIIL000000000).** The genome was assembled in 40 contigs with a total length of 2.44 Mb (N50 = 0.1 Mb) and 41% of G+C content (Tab. S2). The draft genome was quite complete (99.2% of completeness and absence of contamination). In this genome, a total of 2043 genes were annotated including 2007 total CDSs of which 1999 were linked to proteins (Tab. S3). COGs identified mostly fell within functional categories linked to metabolism (50%), information storage and processing (31%), and cellular process and signaling (11%) (Tab. S4, Fig. S2).

The most abundant class of enzymes (ECs) were linked to transferases, hydrolases, and oxidoreductases (Fig. S3).

***Methanobrevibacter arboriphilus* (GenBank: JADIIN000000000).** The genome has been assembled in 83 contigs with a total length of 2.34 Mb (N50 = 44031bp) and 26% of G+C content (Tab. S2). 2043 CDSs were annotated in the genome including 1999 genes coding for proteins identified (Tab. S3). Identified COGs mostly fell within metabolism (48%) and information storage and processing (35%) functional categories (Tab. S4, Fig. S2). The ECs distribution followed the similar pattern reported for *Methanobacterium formicicum* (Fig. S3).

***Aeromicrobium sp.* (GenBank: JADIIM000000000).** The genome was assembled in 60 contigs with a total length of 2.07 Mb (N50 = 60655bp) and 68% of G+C content (Tab. S2). The draft genome has 94.6% of completeness and 1.74% of contamination. A total of 2058 CDSs were annotated, including 2001 CDSs coding for proteins identified (Tab. S3). Identified COGs mostly fell within functional categories linked to metabolism (50.5%), information storage and processing (21%), and cellular process and signaling (21%) (Tab S4, Fig. S2). The most abundant ECs were linked to transferases, hydrolases, and oxidoreductases (Fig. S3).
